# Mechanisms of Chemically Promoted Material Removal Examined for Molybdenum and Copper CMP in Weakly Alkaline Citrate-Based Slurries

**DOI:** 10.3390/ma17194905

**Published:** 2024-10-07

**Authors:** K. U. Gamagedara, D. Roy

**Affiliations:** Department of Physics, Clarkson University, Potsdam, NY 13699-5820, USA; gamageku@clarkson.edu

**Keywords:** chemical–mechanical planarization, tribo-corrosion, tribo-electrochemistry, CMP selectivity, molybdenum in interconnect, diffusion barrier material, removable surface film

## Abstract

Chemical mechanical planarization (CMP) of metal components is an essential step in the fabrication of integrated circuits. Metal CMP is a complex process where strategically activated (electro)chemical reactions serve to structurally weaken the surface layers of the material being processed, and the resulting overburdens are removed under low-force abrasion. Understanding the tribo-electrochemical mechanisms of this process is crucial to successfully designing the consumable materials for advanced CMP slurries that are needed for the new technology nodes. Using a model CMP system involving copper (wiring material in interconnect structures) and molybdenum (a new diffusion barrier material for copper), the present work illustrates a tribo-electroanalytical scheme for studying various mechanistic details of metal CMP. Electroanalytical probes are employed both in the absence and in the presence of surface polishing to quantify the interplay between mechanical abrasion and chemical surface modification. Weakly alkaline slurry formulations are tested with variable concentrations of silica abrasives and a complexing agent, citric acid. The results serve to examine the link between material removal and tribo-corrosion and to identify the functions of the active slurry additives in governing the rates and selectivity of material removal for CMP.

## 1. Introduction

Copper interconnects of integrated circuits (ICs) contain diffusion barriers to prevent migration of copper (Cu) into the dielectrics. The traditional barrier materials (like Ta/TaN) no longer seem suitable for the highly scaled-down new interconnects [1], as the electrical conductivities of ultrathin barriers at the nanoscale drastically decrease due to electron scattering [2]. Only those metals retaining adequate electrical conduction comparable to that of the wiring metal Cu at low dimensions remain useful as diffusion barriers for scaling. The value of the product term, ρ_b_λ, is often used to evaluate a metal’s electrical suitability as a barrier material, where ρ_b_ and λ denote the metal’s bulk resistivity and electron mean free path, respectively. In this approach, metals with ρ_b_λ values less than that of Cu (6.7 × 10^−16^ Ω m^2^) are considered as potential barrier materials, and molybdenum (Mo) is one of the few metals included in this latter category with ρ_b_λ (Mo) = 6.0 × 10^−16^ Ω m^2^ [3].

Mo also has a high melting point (2622 °C), an essential material property to block diffusion of Cu [4]. Moreover, Mo can be used as a substrate for direct electrochemical deposition of Cu, which is essential for filling high-aspect-ratio features during the fabrication of Cu lines [5]. For these reasons, Mo is one of the main candidates currently considered for barrier liners in the new interconnect structures. 

The integration of barrier/Cu lines in the interconnect includes a rigorous sequence of chemical mechanical planarization (CMP) steps, where the bulk Cu is removed, followed by simultaneous planarization of the barrier line with residual Cu. In addition to minimizing CMP-induced surface defects, selectivity of material removal rates (MRRs) between Cu and the barrier material is a critical factor to avoid Cu-dishing as well as excessive loss of barrier material [6]. The strategies required to meet these criteria heavily rely on the selection and functional characterization of the consumables that make up the CMP slurry. The present work centers on these latter aspects of Mo (and Cu) planarization, specifically focusing on the chemical component of CMP and the associated material-functions of a weakly alkaline test slurry. This investigation is motivated primarily by the currently limited availability of published research results involving Mo CMP compared to those of other CMP systems [7,8,9,10,11].

Employing tribo-electroanalytical measurements, we study here the relative roles of silica abrasives and slurry chemicals in governing the overall rates and selectivity of material removal from model CMP samples of Mo and Cu. The experimental slurry is composed of sodium percarbonate (an oxidizer), with or without citric acid as a surface complexing agent, and colloidal silica abrasives. The experiments are designed to probe the chemical component of CMP with and without the incorporation of surface abrasion. The analytical techniques employed in this study involve intermittent (polish vs. hold) open circuit potential (OCP) transient monitoring, electrochemical impedance spectroscopy (EIS), and potentiodynamic polarization. The tribo-(electro)chemical mechanisms of selective material removals from Mo and Cu in the experimental slurries are analyzed. 

## 2. Materials and Methods

The metal test samples (polycrystalline, 99.99% pure Cu, and 99.95% pure Mo, (Kurt Lesker Co., Jefferson Hills, PA, USA), each of 2.54 cm diameter disc, were implanted in Teflon holders equipped with internal metal contacts to support electrical connections. These disc samples, together with their Teflon holders, were attached to a polisher head located in the upper part of a commercial (LaboPol, Struers LLC., Cleaveland, OH, USA) polisher. The experimental CMP slurry had a general formulation of [0.1 M KNO_3_ + 20 mM sodium percarbonate (SPC, Na_2_CO_3_·1.5H_2_O_2_) + x M citric acid (C_6_H_8_O_7_, a complexing agent,) + y wt% colloidal SiO_2_ abrasives (NexSil 125K, Nyacol, Ashland, OH, USA) using chemicals from Fisher Scientific (Pittsburg, PA, USA). Slurry pH was adjusted by using KOH or HNO_3_. The values of x and y were varied in different experiments. Six different slurries based on these variations of x and y were used for each test metal. Detailed compositions of these slurry electrolytes and their ohmic resistances (*R*_s_, measured using EIS) are in Table 1. These slurries also served as the electrolytes for (tribo-)electrochemical measurements. 

All experiments were performed in a tribo-electrochemical test cell combined with a commercial polisher (Struers LaboPol, Krakow, Poland), and this test cell has been described elsewhere in considerable detail [12]. Briefly, this cell allowed in situ electrochemical examination of metal disc samples while the sample surface was subjected to CMP. Each metal sample (separately) served as the working electrode (WE) in a three-electrode configuration with a stainless-steel ring and a saturated calomel electrode (SCE) employed as the counter electrode (CE) and reference electrode (RE), respectively. A polishing pad (IC-1000, Rohm and Haas, Dow Chemical, Newark, NJ, USA) was affixed to the base of a Teflon slurry reservoir attached to the polisher platen. The experiments used two different sample configurations involving stationary hold (H) and dynamic polish (P); in both cases the pad’s pressure on the sample surface was maintained at 0.014 MPa. Electrochemical measurements were carried out using a computer-controlled Solartron 1287 potentiostat (Solartron Analytical–Ametek, Oak Ridge, TN, USA in combination with a 1252 A frequency response analyzer. EIS was performed in the absence of surface abrasion (in slurries at 23 °C), while OCP transients and potentiodynamic data were recorded both in the absence and in the presence of abrasion. 

## 3. Results and Discussion

### 3.1. Ionic Conductivities of Experimental CMP Slurries

**Figure 1:**
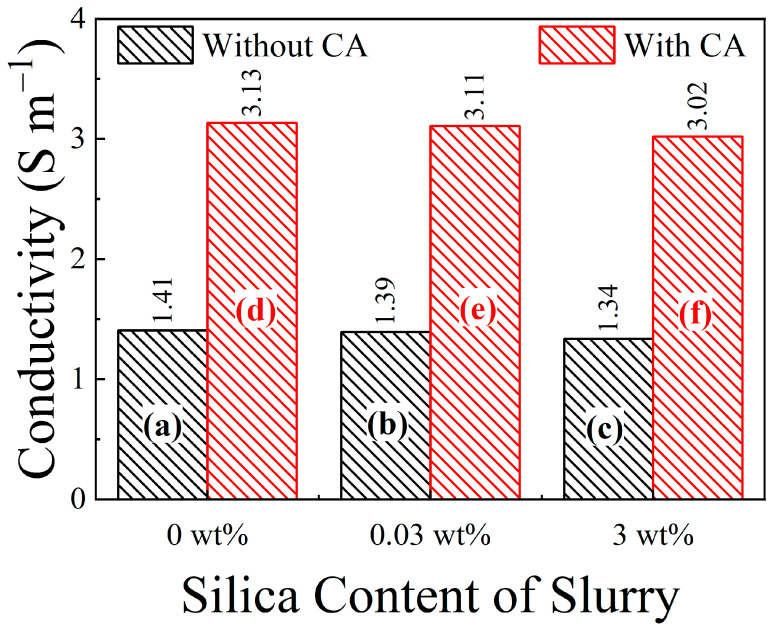
Ionic conductivities of weakly alkaline polishing slurries (at pH = 8) used in the present work. These results show how the presence of 0.1 M citric acid boosts the ionic conductivities of the mixtures of 20 mM SPC and 0.1 M KNO_3_. Bars (a)–(f) represent the congruently labeled slurry solutions listed in Table 1. The numbers associated with the individual bars denote the conductivity values represented by those bars.

Ionic conductivities of the CMP test slurries were measured to ensure that the values were adequate to support the slurries’ function as electrolytes. The ionic strength of CMP slurries is also useful to facilitate the surface reactions that promote MRRs in metal CMP [13]. Conductivity results for the present slurries are shown in Figure 1. The electrical conductivity of 0.1 M KCl solutions (1.29 S m^−1^) at room temperature is often taken as a reference level for ion conduction in electrolytes. The KNO_3_-added slurries examined in Figure 1 clearly meet this conductivity standard even without using the CA additive.

In the present work, the citric acid (CA) complexing agent played an additional role of promoting ionic conduction in the slurry. This is observed in Figure 1, where the slurries including CA consistently exhibit higher ionic conductivities compared to those without CA. The dissolved K^+^ and NO_3_^−^ ions of KNO_3_ support slurry conductivity. CA dissociates as H^+^ and citrate ions, solution-phase transports of which enhance the conductivity of CA-containing slurries. This role of CA as a promoter of ionic conduction in aqueous media has been previously documented in detail [14]. It is also useful to note in Figure 1 that silica abrasives do not have any major effects on the slurry’s conductivity and related chemistry. 

### 3.2. Effects of Silica Abrasives and Citrate Complexing Agent on Material Removal Rates of Mo and Cu

**Figure 2:**
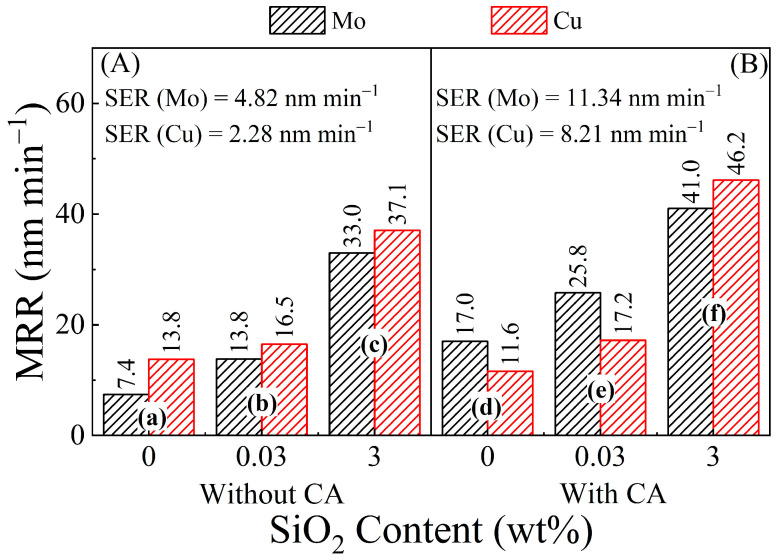
Material removal rates (MRRs) of molybdenum and copper disc samples measured at different concentrations of SiO_2_ abrasives in aqueous mixtures of 20 mM SPC and 0.1 M KNO_3_ at pH = 8, (**A**) with and (**B**) without 0.1 M CA. Bars (a)–(f) represent results for the correspondingly labeled slurry formulations listed in Table 1. The value of MRR denoted by each bar is indicated by the number shown with the bar.

Silica abrasives and citric acid play the leading roles in this work to control, respectively, the mechanical and chemical features of Mo and Cu CMP. Thus, variations in the slurry concentrations of these additives allow for checking two essential aspects of CMP, namely the magnitude and the selectivity of material removal. Figure 2 shows material removal rates (MRRs) of Mo and Cu measured by varying the concentrations of CA and SiO_2_ in the CMP slurries. Static etch rates (SERs) of Mo and Cu coupon samples were measured using abrasive-free slurry solutions that were maintained at 40 °C to approximately simulate the pad temperature under the measurement conditions of MRRs [15,16]. These SER values for slurries (a) and (d) are stated in Figure 2.

The MRRs for the CA-containing slurries are comparable to those reported by other authors for Mo-CMP [7,10,17] and Cu-CMP [18,19,20] using slurry compositions similar to ours (i.e., including silica abrasives and a single oxidizer, with or without a single complexing agent, all in moderate concentrations). In agreement with previously published results, the overall values of MRRs measured here using polycrystalline disc samples are notably lower than those found for deposited thin films [7,9,12,17,21]. These different yields of MRRs are caused by different grain sizes of materials due to the inverse Hall–Petch effect [22,23] and microstructures [24,25] of the two types of test samples. 

To further discuss the observed features of the data in Figure 2, it is useful to consider the following expression of MRRs, which is frequently used to describe the different modes of corrosion- and wear-mediated material removal in metal CMP [26,27]:

*MRR* = *RR*_w_ + *RR*_c_ + *RR*_wc_ + *RR*_cw_ (1)

where *RR*_w_ and *RR*_c_ are the rates of material removal associated with wear alone and corrosion alone, respectively. *RR*_wc_ and *RR*_cw_ denote the rates linked to wear induced corrosion and corrosion-induced wear, respectively. In chemically dominant CMP of metals, a sizable amount of removable material under polishing conditions is generated by corrosion-like reactions of (passive) surface film formation, sometimes coupled with dissolution (dynamic etch). As discussed in the Supporting Material, the aforesaid surface film would generally have a mechanical hardness value considerably less than that of the metal itself and hence would serve as a major component of the removable material under abrasion in CMP. Repeated formation and removal of these films would sustain the planarization process.

As noted above, “corrosion” in metal CMP includes oxidation and complex formation involving chemically passivated surface layers, as well as (usually partial) dissolution of these layers. Thus, the electrochemically measured corrosion rate of a CMP surface during surface polishing can be described as: *CR*(*P*) ≈ *DER* + *CR*_f_, where *DER* and *CR*_f_ denote the rates of dynamic etch and surface film formation, respectively [28]. *DER* takes the value of *SER* under stationary hold conditions, but in general, is not expected to be equal to *SER* due to effects of tribo-corrosion [29]. 

In chemically governed CMP, usually the contribution of *RR*_w_ to the net MRR is small compared to its accompanying terms in Equation (1) and can be neglected in most cases [30]. In this situation, the chemical component of CMP largely originates from the combination [*RR*_c_ *+ RR*_wc_] in Equation (1). The combined value of the latter terms is limited by that of *CR*(*P*), since the removal of the abradable surface film is rate-limited by the film’s formation rate. The deposited film can be entirely removed if the rate of mechanical removal meets the rate of film formation, and this condition is generally maintained under optimized conditions of CMP. In the latter case, Equation (1) can be assumed to have the form:

*MRR* ≈ *f*
*CR*(*P*) + *RR*_cw_ (2)

where *f* is a scaling factor relating the rates of surface film formation and removal. 

The MRRs monitored in metal CMP usually exceed the corresponding values of *CR*(*P*), and this difference can often be accounted for in terms of the contribution of the term, *RR*_cw_, in Equation (2). Finite contributions of *RR*_w_ to the measured MRRs have also been considered in this context [28]. The mechanisms responsible for activating this latter route of material removal can vary among different systems [28,31,32]. The implications of *RR*_cw_ for the present experimental system are further addressed later in the context of the *CR* results. In the following discussion, we examine the chemical and electrochemical reactions that contribute to the *CR* term in Equation (2) to support the MRRs plotted in Figure 2. 

Irrespective of CA’s present inclusion in the CMP slurry, the MRRs of both Mo and Cu consistently increase with increasing silica concentrations. The presence of CA in the slurry increases MRRs of Mo, while the Cu sample also exhibits a similar behavior, especially in the abrasive-containing slurries. The observed role of abrasive particles in material removal has been explained in earlier contact-mechanics models of CMP. For instance, in the framework of Luo and Dornfeld’s model [33], the MRR components included in the framework of Equation (2) should proportionately increase with increasing particle concentrations due to correspondingly increased surface abrasion of the sample. Specifically, the *RR*_cw_ and *RR*_wc_ contributions can be augmented with increasing degrees of abrasion. The [SiO_2_]-dependent trend of MRRs observed in Figure 2 follows the expected outcome of this mechanism. 

For metal planarization, CMP-enabling surface layers of reduced hardness usually form by the anodic components of a mixed reaction, which are supported by the reduction of the slurry’s oxidizer(s). In metal CMP, a cathodic component of oxygen reduction reaction (ORR) is generally present, which, in alkaline slurries, has the form [34],

O_2_ + 2H_2_O + 4e^−^ = 4OH^−^ (3)

where the O_2_ is a dissolved solution species that comes from aerated slurries. In H_2_O_2_-based slurries, the reduction of H_2_O_2_ usually serves as a predominant cathodic step to support corrosion-like anodic reactions:

H_2_O_2_ + 2e^−^ = 2OH^−^ (4)

which represents the alkaline reduction pathway [35]. 

In neutral and alkaline slurries containing H_2_O_2_, MoO_3_ has been found as a predominant oxidation product on the Mo surface [7]. This oxide tends to exist in its hydrated form, MoO_3_·nH_2_O, typically with n ≤ 3 [36]. The value of n (including n = 0) depends on the adsorption characteristics of water and anions at the Mo surface in each slurry. The dihydrate, MoO_3_·2H_2_O (molybdic acid), forms at room temperature [37] and represents a commonly found species of Mo-trioxide hydrates [38]. MoO_3_·2H_2_O forms through an intermediate step of MoO_2_ generation as follows [39]:

Mo + 4OH^−^ = MoO_2_ + 2H_2_O + 4e^−^ (5)

MoO_2_ + 2OH^−^ + H_2_O = MoO_3_·2H_2_O + 2e^−^ (6)

where the right-hand side could be linked to the dehydration of an intermediate species, MoO_3_.3H_2_O [36]. If Equation (4) prevails the Mo sample’s cathodic features, the net mixed reaction of Equations [(4) + (5) + (6)] can be described as:

Mo + 3H_2_O_2_ = MoO_3_·2H_2_O + H_2_O (7)

which may be accompanied by some simultaneous formation of MoO_3_ without hydration. The net rate of these mixed potential reactions that occur during the abrasion of a CMP surface corresponds to the term *CR*(*P*) in Equation (2) and can be estimated from the correspondingly measured corrosion currents. 

In the CA-free slurries at pH = 8, layers of MoO_3_·2H_2_O and MoO_3_ formed on the Mo sample surface should represent the materials for mechanically assisted removal. Additionally, some soluble molybdate ions, MoO_4_^2−^ (a species included in the Pourbaix diagrams of Mo at pH > 6 [40]) can be generated from weak dissolutions of MoO_3_ and molybdic acid: MoO_3_ + 2OH^−^ = MoO_4_^2−^ + H_2_O; and MoO_3_·2H_2_O + 2OH^−^ = MoO_4_^2−^ + 3H_2_O. The SER values stated in Figure 2 for Mo in the CA free solution are most probably supported by the production of these MoO_4_^2−^ ions. Such soluble products of Mo oxidation can form (at their *DER* during polishing) within porous structures of the surface oxide/complex films to further reduce the latter’s hardness for easy removal under low pressure. 

A survey of the literature on Mo-citrate complexes in aqueous media indicates that MoH_−1_Cit(OH)_2_ is the most likely complex that forms in the CA-containing CMP slurries as a removable material on Mo [41]. This complex in the present experiments can result from a chemical reaction between molybdic acid and adsorbed Cit^3−^ anions:

MoO_3_·2H_2_O + Cit^3−^ = MoH_−1_Cit(OH)_2_ + 3OH^−^ (8)

and this Mo-citrate is stable over a broad pH range (pH = 2–11). Since the MRRs of Mo displayed in Figure 2 increase by adding CA to the CMP slurry, it is evident that the structural hardness of the MoH_−1_Cit(OH)_2_ surface film (possibly mixed with molybdic acid) formed on Mo in the presence of CA is lower than that of a molybdic acid layer alone. An active role of SiO_2_ abrasives in material removal is observed in agreement with earlier reports of Mo CMP where SiO_2_ has been used in H_2_O_2_-based slurries [9,21,42]. The comparatively higher value of SER (Mo) noted in Figure 2 for the CA-containing solution (d) can be attributed to the weak dissolution of MoH_−1_Cit(OH)_2_. 

The effect of SiO_2_ on the MRRs for Cu in Figure 2B is like that seen for Mo in Figure 2A. The effects of CA on MRR (Cu) are also mostly comparable to those manifested for MRR (Mo), but in a relatively less pronounced form. In the absence of CA, the Cu test sample supports Cu_2_O and Cu(OH)_2_ as the primary surface species produced in the following anodic steps [43,44,45]:

Cu + 2OH^−^ = Cu(OH)_2_ + 2e^−^ (9)

2Cu + 2OH^−^ = Cu_2_O + H_2_O + 2e^−^ (10)

where the reduction of H_2_O_2_ serves as a predominant interfacial source of OH^−^ that is needed for reactions (8)–(10) to operate in a mixed potential mode. The mixed potential forms of reactions [(4) + (9)] and [(4) + (10)] can be expressed, respectively, as follows:

Cu + H_2_O_2_ = Cu(OH)_2_ (11)

2Cu + H_2_O_2_ = Cu_2_O + H_2_O (12)

where Cu(OH)_2_ and Cu_2_O serve as the main constituent materials of the mechanically removable surface layers of chemically modified Cu. Some surface species of CuO likely form to accompany the reactants in Equations (11) and (12) [43]: Cu(OH)_2_ = CuO + H_2_O. The relatively low value of SER (Cu) noted in Figure 2 for the CA-free solution can be associated with small amounts of CuOH^+^ ions present in the system [34]: Cu^2+^ + H_2_O = CuOH^+^ + H^+^.

At the slurry pH of 8, Cit^3−^ (Cit ≡ C_6_H_5_O_7_) is the predominant species of CA; this is illustrated in a speciation diagram for CA in Figure S1 of the Supplementary Material. In the abrasive-free case, addition of CA to the H_2_O_2_-based slurry slightly reduces the MRR of Cu, which can happen due to competitive adsorptions of Cit^3−^ and H_2_O_2_ at the surface sites of Cu. In the CA containing slurries at pH = 8, the predominant Cu-citrate species is (Cu_2_Cit_2_H_−2_)^4−^, which can result from the following reactions of the surface species, Cu_2_O, CuO, and Cu(OH)_2_ [46]:

Cu_2_O + 2Cit^3−^ = (Cu_2_Cit_2_H_−2_)^4−^ + H_2_O + 2e^−^ (13)

2Cu(OH)_2_ + 2Cit^3−^ = (Cu_2_Cit_2_H_−2_)^4−^ + 2H_2_O + 2OH^−^ (14)

2CuO + 2Cit^3−^ = (Cu_2_Cit_2_H_−2_)^4−^ + 2OH^−^ (15)

where reactions (14) and (15) are chemically activated without involving interfacial charge transfer; reaction (13) can occur in a mixed mode with reaction (3) as: (1/2)O_2_ + Cu_2_O + 2Cit^3−^ = (Cu_2_Cit_2_H_−2_)^4−^ + 2OH^−^; or, with reaction (4): Cu_2_O + H_2_O_2_ + 2Cit^3−^ = (Cu_2_Cit_2_H_−2_)^4−^ + 2OH^−^ + H_2_O.

The increased value of SER (Cu) detected with the inclusion of CA in the slurry can be associated with the soluble species (CuCit_2_H_−2_)^4−^, released from surface films of Cu_2_O and Cu(OH)_2_ [46]. This inference was confirmed by the bluish-green color of Cu-citrate that developed in the slurry solution used for SER testing. Partial dissolution of the oxide/hydroxide layers of Cu in the form of (CuCit_2_H_−2_)^4−^ (occurring at the *DER* during surface abrasion) mechanically weakens the oxidized surface layers of Cu under low-pressure abrasion. Co-adsorption of SiO_2_ can stabilize the adsorption of H_2_O_2_ on Cu [47]. The MRR of Cu moderately increases as the slurry’s silica content is increased from 0.03 to 3 wt%. This increase in Cu removal occurs from a combined action of mechanical abrasion and chemical formation of (CuCit_2_H_−2_)^4−^. 

### 3.3. Effects of Silica Abrasives and Citrate Complexing Agent on the Strength and Selectivity of Material Removal from Mo and Cu

As seen in Figure 2, MRRs of both Mo and Cu continue to increase with increasing concentrations of SiO_2_ in the slurry. However, these relative increments are different for the two metals, which suggests that the chemically dictated material selectivity of CMP may be affected under increased mechanical abrasion. The results presented in this section focus on these relative roles of absolute and relative (selective) MRRs. While the reported values of [Barrier:Cu] CMP selectivity vary between ~0.3 and ~3.0 [48,49,50,51], to avoid Cu dishing and loss of barrier lines, this selectivity is often chosen to be at or near the ratio, 1:1 [50,52]. However, slurry designs aimed at controlling CMP selectivity largely rely on the chemical component of CMP, and these selectivity values are set in most cases using MRR data for blanket wafers of the individual metals. 

**Figure 3:**
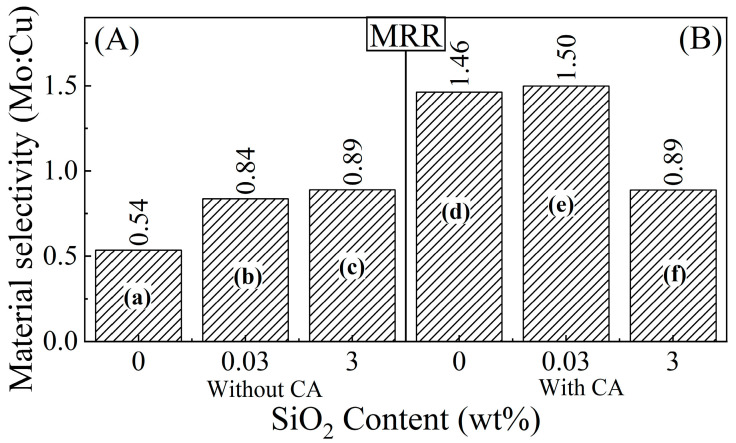
Selectivity (Mo:Cu) of material removal compared between the CMP test samples of Mo and Cu. Bars (a)–(f) represent results for the correspondingly labeled polishing slurries (a)–(f) in Table 1. The slurries contain (**A**) 0 M or (**B**) 0.1 M CA. The selectivity values (identified by the numbers above the individual bars) represent the ratio [MRR (Mo):MRR (Cu)] calculated using the data from Figure 2.

Since the polishing pressure is not uniform at different regions of an IC pattern, preset selectivity values based on blanket materials can considerably change when a patterned wafer is polished [53]. Due to this reason, it is useful to maintain a sufficient window of selectivity while determining its target values with blanket wafers or disc samples of single metals. Based on these considerations, the experimental slurries for this work were intended to yield Mo:Cu selectivity in the range 1 ± 0.5. These selectivity values, as determined using the MRR data from Figure 2, are shown in Figure 3. 

With increased abrasive contents of a CMP slurry, the number of abrasive particles situated at the sample-pad contact regions (“active” abrasives) increases, which in turn augments the mechanical action of CMP. At 0 and 0.03 wt% abrasive concentrations, selectivity and strength of material removal largely share the same increasing trend following the abrasive’s inclusion in the slurry. By adding 3 wt% abrasives to the slurry, the Mo:Cu CMP selectivity only slightly increases in the CA-free slurries but decreases in the CA-based slurries. In contrast, the absolute values of MRRs for both Mo and Cu continue to increase with increasing [SiO_2_], both in the absence and in the presence of CA. 

The chemically modified material of Mo for mechanical removal is considered here as composite surface films of [MoO_3_/MoO_3_·2H_2_O] and [MoH_−1_Cit(OH)_2_] with and without CA in the slurry, respectively. For Cu, these CMP surface films are composed of [Cu_2_O/Cu(OH)_2_]/CuO with and without embedded species of (CuCit_2_H_−2_)^4−^ in the CA-containing and CA-free slurries, respectively. Abrasion of SiO_2_ facilitates the removal of these materials through the mechanisms associated with the terms *RR*_c_ and *RR*_cw_ in Equation (2). 

The contributions of *RR*_cw_ to the measured MRRs are most likely affected by the metals’ crystallographic properties [28] and hence yield different values for Mo and Cu. Since the CMP selectivity is determined as ratios of MRRs (instead of absolute MRRs), the [SiO_2_]-dependent trend of selectivity values correspondingly deviates from that of absolute MRRs. Although the mechanism of *CR*(*P*) generally plays a major role in dictating the CMP selectivity of metal-slurry systems, the relation between *CR*(*P*) and *RR*_cw_ is system-dependent [32]. As a result, the contribution of the latter to the measured MRR may not follow the chemically designed CMP selectivity of *CR*. 

The degree of decoupling between *RR*_cw_ and the main chemical effects (*RR*_c_) of CMP is expected to increase as the mechanical component is reinforced, such as by increasing abrasive concentrations. Additionally, increased mechanical action of CMP can also activate a measurable component of *RR*_w_ in Equation (2), which would be relatively insensitive to chemical changes of slurry compositions that affect CMP selectivity [28]. Signature features of this latter situation are seen in Figure 3B, where the [Mo:Cu] CMP selectivity drops by increasing the slurry’s [SiO_2_] from 0.03 wt% to 3 wt%, while the MRRs of both Mo and Cu in the same transition increase in Figure 2B. Since the overall MRRs measured in the absence of CA are lower than those found in the presence of CA, it is likely that the aforesaid roles of *RR*_cw_ remain relatively suppressed unless the topmost surface layers are structurally altered by complex formation with CA. In the latter case, the results for CMP selectivity in Figure 3A mostly follow the trend of MRRs in Figure 2A. This effect is further examined later in this report by comparing MRRs with electrochemically controlled tribo-corrosion rates (TCRs).

### 3.4. Chemical Mechanism of Mo CMP Examined Using Impedance Spectroscopy

Electric equivalent circuit (EEC) models of electrochemically active CMP interfaces obtained by EIS can provide an overall description of the reaction mechanisms operated at those interfaces. Changes in the EEC elements introduced by changing experimental control variables can be analyzed to obtain additional kinetic details about the associated surface reactions. If a CMP interface supports EIS measurements under polishing, it is also possible to examine how the reactions are affected by tribology. For most CMP systems, however, it is difficult to collect EIS data while meeting all the validation criteria for EIS [54]. Although the EIS results of the present investigation are in the latter category, they serve as a useful tool to check if the impedance characteristics of the CMP test systems are consistent with the reaction mechanisms considered in Equations (3)–(15). 

EIS data were recorded at the stabilized OCPs using alternating current (AC) perturbation spectra of 10 mV average amplitude and frequencies ranging from 1 Hz to 10 kHz in logarithmically spaced intervals. The low frequency range of EIS was selected to ensure that any residual drifts of the OCP were substantially slower than the lowest frequency of AC perturbation. The high frequency range of EIS was restricted by the requirement of avoiding inductive effects of electrical leads and connectors. The sample and the polishing pad were in stationary contact at a pressure of 0.014 MPa. 

**Figure 4:**
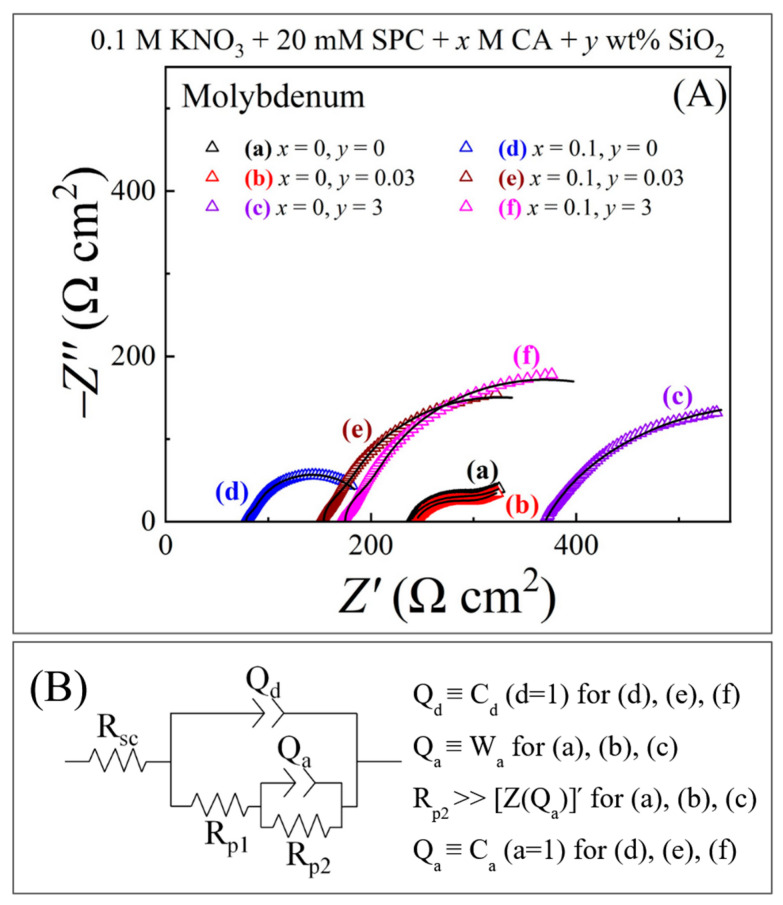
(**A**) Nyquist impedance plots recorded under the stationary hold condition of a Mo test sample that was maintained at its OCP with a pad-sample contact pressure of 0.014 MPa in different polishing slurries. Plots (a)–(f) represent the correspondingly labeled slurries in Table 1. The symbols are data points, and the associated lines are CNLS fits to the data based on the circuit model shown in panels (**B**). The circuit elements are discussed in the main text.

Figure 4A shows Nyquist impedance plots for the Mo sample recorded at OCPs using EIS in the six test slurries Z′ and Z″ denote, respectively, the real and imaginary parts of the system’s complex impedance, Z. The symbols and the lines represent experimental data and complex nonlinear least squares (CNLS) fits to the data, respectively. With trial CNLS calculations, a generalized EEC, shown in Figure 4B, was found to fit all six Nyquist plots in Figure 4A; some adjustments in the makeups and values of the constituent impedance elements were necessary as annotated beside the circuit model in Figure 4B. 

In Figure 4B, *R*_sc_ is the slurry resistance measured in the contact mode under a pad-sample pressure of 0.014 MPa. *Q_d_* denotes a constant phase element (CPE), which is a frequency-dispersed version of the double layer capacitance. The CPE, *Q*_a_, is linked to adsorption of anions such as OH^−^ as well as Cit^3−^. The impedance, *Z*(*Q*_d_), of *Q_d_* is defined as:

*Z*(*Q*_d_) = 1/[*Y*_d_(*jω*)^d^] (16)

where *Y*_d_ and *d* are frequency-independent CPE parameters for the double layer, and *ω* is the perturbation frequency of EIS. Similarly, the impedance of Q_a_ is: *Z*(*Q*_a_) *= 1*/[*Y*_a_(*jω*)^a^], with *Y*_a_ and *a* denoting the CPE parameters linked to anion adsorption. 

The polarization resistance, *R*_p1_, can be taken as an equivalent parallel combination of two charge transfer resistances for reactions (4) and (5). Likewise, the polarization resistance, *R*_p2_, can be attributed to reactions (4) and (6). For plots (a), (b), and (c), CNLS analyses did not yield a reliable (low error) value of *R*_p1_, and hence, suggested that *R*_p1_ was large compared to the real part of the Warburg impedance within the experimental frequency range of EIS. As a result, the perturbation current through *W*_a_ shunted out *R*_p1_ from the [Z(*W*_a_) − *R*_p1_] parallel combination that was subjected to CNLS detection. The CNLS-calculated values of the EEC elements from Figure 4B are summarized in Table 2. 

Among the EEC variables listed in Table 2, *R*_sc_, *Y*_d_, and *R*_p1_ are detected for all six slurries and are chiefly relevant for checking the CMP chemistries of Mo described in Equations (4)–(8). *R*_sc_ represents a pad-sample *contact resistance* measured in the polishing slurry. This resistance is specific to a metal-pad CMP interface, and its value generally is large compared to that of the usual slurry (solution) resistance, *R*_s_ (listed in Table 1), measured without the presence of a polishing pad pressed against the metal sample’s surface. The origin of *R*_sc_ and its connection with *R*_s_ are discussed in detail in the Supplementary Material. Due to a relatively higher ionic conductivity of the Cit^3−^-containing slurries, their *R*_s_ values decrease in the presence of CA (in Table 1), and the corresponding *R*_sc_ values also drop below those of the CA-free slurries (in Table 2). 

In slurries, (a), (b), and (c), *Y*_d_ increases as the SiO_2_ concentration is increased to 3 wt%. This observation can be linked to the known interactions of SiO_2_ with water molecules [55], because the surface layer of molybdic acid formed on Mo is rich in its water content. At high densities of SiO_2_ adsorbed at the Mo surface, EIS senses the water component of MoO_3_·2H_2_O interacting with SiO_2_ as a capacitor in parallel with the intrinsic double layer capacitor of the Mo surface, and this results in the increased value of *Y*_d_ found at the slurry’s 3 wt% SiO_2_ content. 

The relatively smaller values of *Y*_d_ observed for slurries (d), (e), and (f) indicate that the presence of CA in these cases changes the chemical makeup (and the interactions) of the Mo sample’s surface layer. Equation (8) accounts for these latter changes in terms of Cit^3−^-mediated conversion of molybdic acid to the Mo-citrate complex, MoH_−1_Cit(OH)_2_. The chemical reaction (8) does not contribute a separate impedance element to the polarization branch of the EEC. However, reaction (8) reduces the Mo surface layer’s passivating strength, reflected in the value of *R*_p2_, which is seen to drop in the transition from the slurries without CA to those with CA. The values of both *R*_p1_ and *R*_p2_ increase with increasing [SiO_2_] in the slurry as the site-blocking abrasive adsorbates reduce the fraction of the Mo surface available to support charge transfer reactions. The value of *C*_a_ decreases as [SiO_2_] increases since the area dependence of a capacitance is inverse of that for a resistance. 

### 3.5. Chemical Mechanism of Cu CMP Examined Using Impedance Spectroscopy

**Figure 5:**
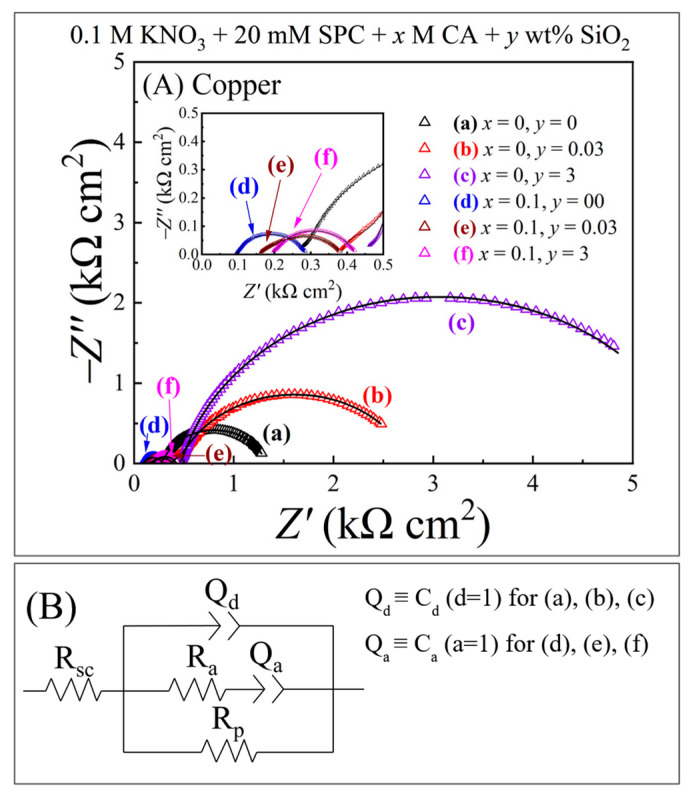
(**A**) Nyquist impedance plots collected under the stationary hold condition of a Cu test sample that was maintained at its OCP with a pad-sample contact pressure of 0.014 MPa in different polishing slurries. Plots (a)–(f) are for the correspondingly labeled slurries in Table 1. The symbols are data points and the lines through the symbols are CNLS fits to the data based on the circuit model shown in panels (**B**). The circuit elements are discussed in the main text.

Figure 5A presents Nyquist impedance plots for the Cu sample recorded at OCPs, with the sample–pad interface maintained at a pressure of 0.014 MPa under stationary conditions. The symbols and the lines correspond to experimental data and CNLS fits to the data, respectively. The inset shows the high-frequency region of the plots. By comparing the impedance scales in Figure 4A and Figure 5A, it can be noted that the overall impedance of this Cu CMP system is about an order of magnitude higher than that of the Mo system, especially in the CA free slurries. This passivating nature of the Cu surface is manifested in the relatively large Nyquist arcs of plots (a), (b), and (c) in Figure 5A, where in the absence of CA, the sample surface is populated by relatively passive species of Cu-hydroxide and -oxides.

The progressive expansion of the Nyquist arcs from plots (a) to (c) in Figure 5A shows how the addition of silica to the slurry enhances the Cu sample’s surface impedance. This site-blocking effect of silica on Cu is essentially the same as that observed for the Mo sample in Figure 4A. With the addition of CA to the slurry, the diameters of the Nyquist arcs (d), (e), and (f) shrink as the Cu sample’s surface impedance drops. 

With increasing [SiO_2_], *R*_sc_ increases, mostly due to the site-blocking nature of adsorbed abrasives. Reactions (13)–(15) activated by citrate ions in the slurry weaken the structural integrity of the Cu-hydroxide/oxide surface film. Consequently, plots (d), (e), and (f) in Figure 5A are considerably shrunken compared to their corresponding plots (a), (b), and (c). 

Figure 5B presents the general EEC model for the Cu-CMP interface obtained by CNLS fitting the EIS data in Figure 5A. This modified Randles EEC is determined chiefly by the intrinsic aqueous electrochemistry of Cu and is commonly found in the literature of Cu CMP. Here *R*_sc_, *Q*_d_ (or *C*_d_), and *Q*_a_ (or *C*_a_) have the same general meanings as those of their counterparts in Figure 4B. *R*_p_ in Figure 5B represents the experimental system’s net polarization resistance, which is dominated by reactions (11) and (12) in the absence of CA and by reactions (11)–(15) in the presence of CA. The inclusion of a nonfaradaic (*R*_a_-*Q*_a_) impedance branch in Figure 5B is based on the well-known affinity of Cu electrodes for anion adsorptions that mostly operate in the nonfaradaic mode under OCP conditions [45,56,57]. Two special cases of the general EEC in Figure 5B emerge due to system-specific variations in the spatial inhomogeneity and roughness of the Cu surface; these special cases are noted next to the EEC schematic in Figure 5B.

The full set of CNLS calculated variables from Figure 5B are listed in Table 3 for completeness. Here, the values of *R*_p_ and *R*_sc_ are most pertinent to checking the reaction mechanisms of CMP. As the slurry’s SiO_2_ content is increased, the value of *R*_p_ exhibits a parallel increase, which can be attributed once again to blocking of electrochemically operative surface sites by abrasive particles. According to the observed values of *R*_p_ in Table 3 (and the radii of the Nyquist arcs in Figure 5A), the Cu surface, being populated by adsorbed citrate ions in this situation, has a comparatively lower impedance. This Cu surface also exhibits a capacitive feature of spatially uniform adsorption instead of a CPE. The values of *R*_sc_ in Table 3 exhibit essentially the same trends with variations of [SiO_2_] and [CA] as those seen for *R*_sc_ in the case of Mo in Table 1. 

In view of the foregoing discussions of Figure 4 and Figure 5, the impedance data recorded under stationary conditions of the Mo and Cu test systems are overall consistent with the reaction mechanisms described in Equations (3)–(15). According to the currently available electrochemical and tribo-electrochemical results for metal CMP systems, the same chemical mechanisms of CMP largely operate in the presence of mechanical surface abrasion, while the rates of the different (electro)chemical steps are selectively or collectively affected [12,58,59]. Thus, the EIS results in Figure 4 and Figure 5 provide a phenomenological basis to utilize these reaction mechanisms for further characterizations of the CMP systems under polishing conditions. 

### 3.6. Manifestation and Detection of CMP Enabling Tribo-Electrochemistry

Surface complex films formed as removable materials on metals for CMP generally exhibit different levels of anodic and cathode activities compared to those of their underlying pure metals. Thus, if a metal surface exhibits selective suppression or elevation of its anodic or cathodic activity after undergoing CMP related chemical modifications, these changes in the metal’s faradaic selectivity can be electrochemically probed and used as an overall efficiency indicator of the CMP-enabling surface chemistries. Intermittent OCP transients, measured with alternated activation and deactivation of surface abrasion, are a widely used tool for these measurements [12]. 

The basic working principle of the intermittent OCP transient method for checking chemical efficacies of CMP systems can be elaborated using the following mixed potential expression of the OCP (*E*_OC_) for a metal supporting a corrosion-like process [12,60,61]:

(17) $${E_{\mathrm{OC}}\equiv E}_{\mathrm{corr}}=B_{0}\left(\frac{E_{\mathrm{ra}}}{\beta_{a}}+\frac{E_{\mathrm{rc}}}{\beta_{c}}\right)+B_{0}\ln\left(\frac{i_{\mathrm{rc}}A_{c}}{i_{\mathrm{ra}}A_{a}}\right)$$

where the theoretical equivalence between *E*_OC_ and *E*_corr_ is explicitly noted. *β*_a_ and *β*_c_ are the symmetry factors of the anodic and cathodic steps constituting the mixed potential system; *i*_rc_ and *i*_ra_ are the corresponding exchange current densities; *E*_rc_ and *E*_ra_ denote the equilibrium Nernst potentials of the associated cathodic and anodic steps, respectively. $\beta_{a}=RT/[zF(1\;-{\;\alpha }_{a})]$ and $\beta_{c}=RT/(zF\alpha_{c})$, with *R*, *F*, and *T* denoting the gas constant, Faraday constant, and ambient temperature, respectively. $\alpha_{a}$ and $\alpha_{c}$ are the anodic and cathodic transfer coefficients, respectively; *z* is the electron valency of the mixed reaction; *B*_0_ = *β*_c_ *β*_a_/(*β*_c_ *+ β*_a_). The partial surface areas of the metal sample supporting cathodic and anodic processes are denoted as *A*_c_ and *A*_a_, respectively. 

The terms ($i_{\mathrm{rc}}A_{c}$) and ($i_{\mathrm{ra}}A_{a})$ in Equation (17) are often considered as the primary indicators of the cathodic and anodic activities of a CMP interface, respectively [62,63,64]. The CMP specific surface film of a metal sample can be grown to a saturation thickness by holding the sample stationary under the typical polishing pressure of a pad in the experimental slurry. Complete removal of the film by a subsequent polishing step can generate the surface conditions of a post-CMP, bare metal surface. Comparative values of the OCPs measured under stationary hold (H) and active polish (P) will indicate how the ratio, [($i_{\mathrm{rc}}A_{c}$)/($i_{\mathrm{ra}}A_{a})$], of the test metal’s cathodic and anodic activities are affected in going from full surface coverage (in H) to zero (or nearly zero) coverage (in P) of the complex film. 

In CMP-related treatments of the Nernst equation, it is customary to treat the activity terms of all solid phases on the sample surface with a value of unity [65]. In this formulation, the values of *E*_rc_ and *E*_ra_ for a bare metal should not be affected by a complex film formed on the metal. Additionally, corresponding variations in the values of *α*_c_ and *α*_a_ in most cases are found to be insignificant to measurably affect those of *E*_OC_ in Equation (17). If these conditions apply to the CMP system studied here, it is possible to write:

(18) $$E_{\mathrm{OC}}\left(H\right)-E_{\mathrm{OC}}\left(P\right)\approx B_{0}\ln\left(\frac{X_{c}}{X_{a}}\right)$$

where *E*_OC_ (H) and *E*_OC_ (P) are OCPs of the CMP interface measured at a certain instant during the “hold” and “polish” stages, respectively; *i*_rc_ (H) [or *i*_ra_ (H)] and *i*_rc_ (P) [or *i*_ra_ (P)] denote the exchange currents of the constituent cathodic (or anodic) reactions of mixed potential equilibrium under hold and polish conditions, respectively. *X*_c_ and *X*_a_ denote, respectively, the strengths of anodic and cathodic selectivity at the chemically modified CMP surface; *X*_c_ = [$\left(i_{\mathrm{rc}}\left(H\right)A_{c}\left(H\right)\right)$/$\left({i_{\mathrm{rc}\;}(P)\;A}_{c}\left(P\right)\right)$] and *X*_a_ = [$\left(i_{\mathrm{ra}}\left(H\right)A_{a}\left(H\right)\right)$/$\left({i_{\mathrm{ra}}(P)\;A}_{a}\left(P\right)\right)$]. 

The phenomenological framework of Equation (18) can be used to assess the chemical efficacy of a CMP process in terms of the CMP-enabling surface complex film’s faradaic selectivity, and this can be accomplished by measuring the difference, [*E*_OC_ (H) − *E*_OC_ (P)], of OCPs supported under the sample’s hold and polish conditions. For instance, if the cathodic activity of the CMP sample’s surface selectively increases (or the anodic activity selectively decreases) due to the deposition of a complex/oxide surface film, then *X*_c_ > *X*_a_ and *E*_OC_ (H) − *E*_OC_ (P) > 0 in Equation (18). In the opposite situation, where the sample surface becomes anodically more active than its cathodic function in the presence of a surface film, then *X*_c_ *< X*_a_ and *E*_OC_ (H) − *E*_OC_ (P) < 0 in Equation (18). 

According to the above discussion, the sign of the OCP difference, [*E*_OC_ (H) − *E*_OC_ (P)], can indicate the faradaic function of a CMP-enabling surface layer formed in a slurry. This information can be useful to analyze the chemical mechanism of the associated CMP process and hence, to tune the chemical formulation of the CMP slurry. At the same time, the magnitude of [*E*_OC_ (H) − *E*_OC_ (P)] can be used as an empirical measure of a chemically dominated CMP system’s tribo-electrochemical functions. In general, an appreciable finite value of | *E*_OC_ (H) − *E*_OC_ (P)| should be essential to supporting measurable rates of material removal in CMP [60]; these points are elaborated in the next section with experimental data.

### 3.7. Tribo-Electrochemical Efficiencies of CMP for Mo and Cu Probed Using Intermittent OCP Transients

**Figure 6:**
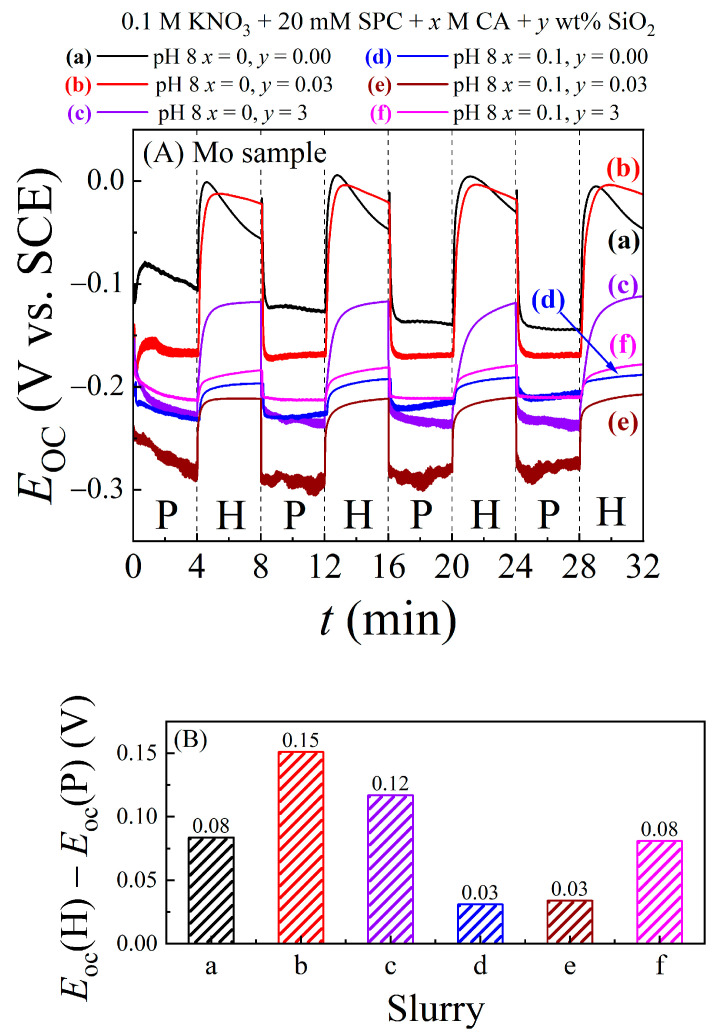
(**A**) Intermittent OCP transients recorded in alternated cycles of polish (P) and hold (H) using a Mo CMP sample in different polishing slurries. The plots have been labeled according to their representative slurries, (a)–(f). The sample-pad pressure was maintained at 0.014 MPa while the P and H sequences were repeated in 4 min intervals. Panel (**B**) shows the slurry-specific difference-OCPs with their individual values stated above the corresponding bars. The results in (**B**) were obtained using the *E*_OC_ data from panel (**A**).

Figure 6A shows intermittent OCP transients for the Mo sample recorded in the CMP slurries (a)–(f). The sample surface was subjected to polish (P) and hold (H) conditions in alternated 4 min cycles with the Mo surface maintained in its contact mode with the polishing pad at a pressure of 0.014 MPa throughout the full experiment. The mostly repeatable OCP features observed in the successive H (as well as P) cycles demonstrate that the slurry is stable with respect to repeated P/H cycles and that the Mo surface does not undergo any significant irreversible changes as these cycles are operated. 

In most of the P segments and in some of the H segments, the *E*_OC_ values are mostly time independent. Under these steady-state conditions of polishing, the growth and removal of the surface complex are largely balanced. Time invariant values of *E*_OC_ in the H sequences imply completion of saturation surface coverage by the complex/oxide species, and this situation is mainly found for the CA-containing slurries. In the CA-free slurries, *E*_OC_ generally continues to vary, indicating incomplete stabilization of the surface complex layers within the sampling times. The *E*_OC_ (P) data show the expected signature of tribo-noise, the strength of which is system dependent, as discussed elsewhere [12]. 

The values of *E*_OC_ averaged separately from the final 1 min segments of the H and P cycles are used, respectively, as representative experimental values of *E*_OC_ (H) and *E*_OC_ (P) in Equation (18). These averaged data points are used to plot the bar graphs of the slurry-dependent difference OCPs [*E*_OC_ (H) − *E*_OC_ (P)] in Figure 6B. According to the discussion of Equation (18), the positive values of [*E*_OC_ (H) − *E*_OC_ (P)] imply that the complex/oxide surface species formed on the Mo test sample are anodic inhibitors and/or cathodic promoters. As demonstrated in the next section, the specific nature of the CMP sample’s electrochemical activity can be determined using potentiodynamic measurements. 

**Figure 7:**
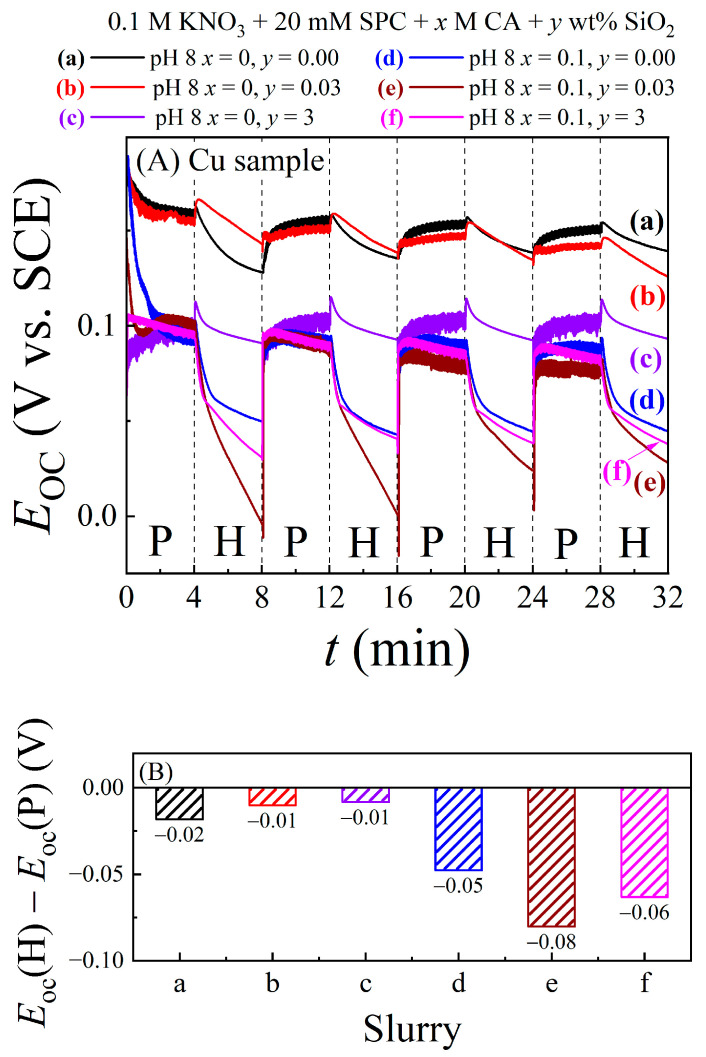
(**A**) Intermittent OCP transients recorded in alternated cycles of active polish (P) and stationary hold (H) using a Cu CMP sample in different polishing slurries. Plots (a)–(f) correspond to the slurries, (a)–(f), listed in Table 1. The Cu sample was pressed down on to the pad at 0.014 MPa under both the hold and polish conditions, and the P and H sequences were repeated in 4 min intervals. Panel (**B**) shows the slurry-specific difference-OCPs with their individual values stated above the corresponding bars. The results in (**B**) were obtained using the *E*_OC_ data from panel (**A**).

Figure 7A displays intermittent OCP transients for the Cu CMP sample, recorded in the same way and using the same slurries as those employed for Mo in Figure 6. The values of [*E*_OC_ (H) − *E*_OC_ (P)] for Cu were determined using the averaging procedure mentioned in the context of Figure 6. These difference OCPs for Cu are shown in Figure 7B. The relatively time-independent nature of the *E*_OC_ in the polish segments indicates a steady state between chemical formation and mechanical removal of surface layers. In the hold stages, however, the *E*_OC_ plots in Figure 7A exhibit notable time dependencies, indicating continued changes in the amount and/or composition of the Cu surface. Unlike the case of Mo, *E*_OC_ (P) > *E*_OC_ (H) for Cu in all the six slurries used. Thus, the difference OCPs in Figure 7B have negative signs, which, according to Equation (18), imply suppressed cathodic activities and/or enhanced anodic activities of the Cu surface films. Specific details of this latter observation are further addressed in the next section.

### 3.8. Results of Tribo-Potentiodynamic Polarization Measurements 

**Figure 8:**
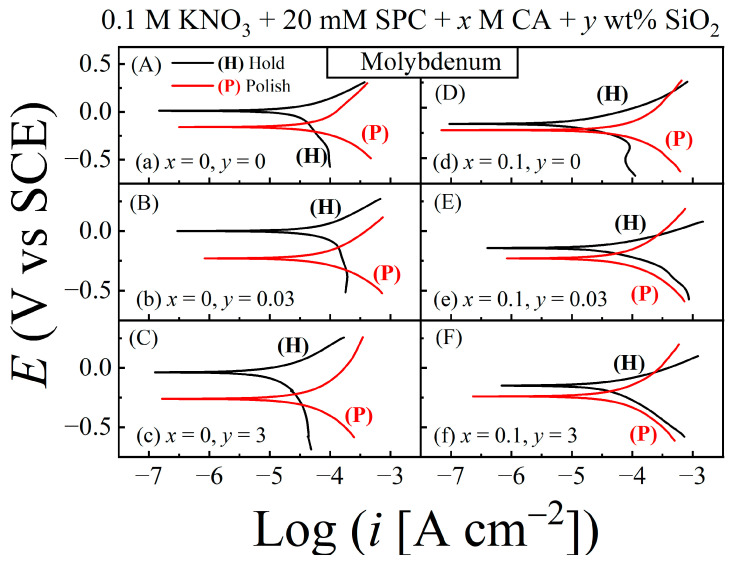
Potentiodynamic polarization plots for a Mo sample compared between the conditions of hold (H) and polish (P) using the different slurries. Panels (**A**–**F**) display the results for different combinations of x and y, the concentrations of CA and SiO_2_, respectively. These combinations are indicated in the individual panels using the slurry-labeling schemes (a)–(f) from Table 1. The upper and lower branches of each plot represent activated anodic and cathodic reactions, respectively, while the intersection point of the two branches appears at *E* = *E*_corr_ along the potential axis. The sample–pad contact was maintained at a down-pressure of 0.014 MPa in both hold and polish modes. All plots were corrected for the ohmic drop of solution resistance.

**Figure 9:**
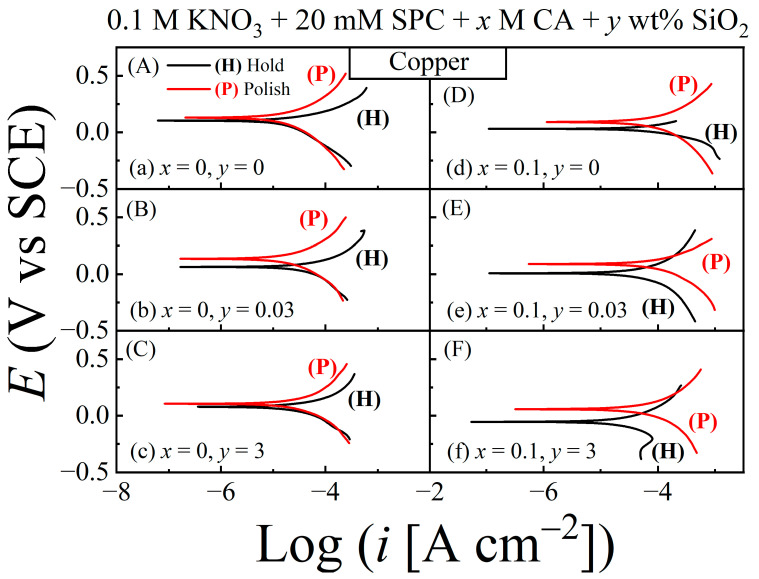
Potentiodynamic polarization plots for a Cu sample compared between the conditions of (a) stationary hold (H) and (b) active polish (P) using different slurries. Panels (**A**–**F**) show the results for different combinations of x and *y*. These combinations for the individual panels are indicated using the slurry-labeling schemes (a)–(f) introduced in Table 1. The test conditions for Cu and the arrangements of the plots in the different panels in Figure 9 are the same as those considered in Figure 8 for the Mo sample.

**Figure 10:**
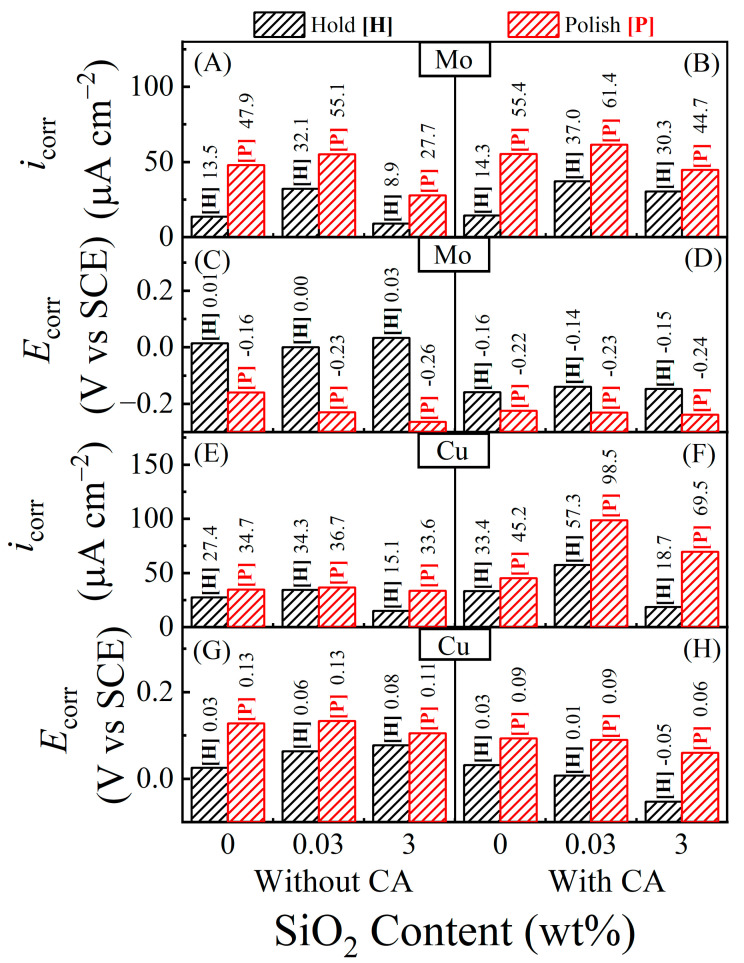
Corrosion current densities [(**A**,**B**,**E**,**F**)] and corrosion potentials [(**C**,**D**,**G**,**H**)] for Mo [(**A**–**D**)] and Cu [(**E**–**H**)] test samples, obtained from the potentiodynamic data in Figure 8 (for Mo) and Figure 9 (for Cu). The data panels arranged in the left and right columns correspond to the experimental CA-free and CA-containing slurries, respectively. The number next to each bar represents the value of the quantity plotted with that bar. Polish and hold conditions of the experiments are indicated, respectively, by the labels [H] and [P] associated with the bars. The data in [(**A**,**C**,**E**,**G**)] and those in [(**B**,**D**,**F**,**H**)] are for the slurries containing 0.0 and 0.1 M CA, respectively.

Potentiodynamic polarization plots were recorded using linear sweep voltammetry (LSV) at a 5 mVs^−1^ rate of potential scan in the direction of increasing voltages and typically exploring a voltage range of *E*_OC_ ± 0.5 V. Results for the Mo and Cu CMP samples are presented in Figure 8 and Figure 9, respectively. The plots recorded under hold and polish conditions are labeled (H) and (P), respectively. The different slurry compositions are noted in their corresponding data panels. While these data are organized to compare the polarization features of growth (in hold) vs. removal (in polish) of surface films, a different arrangement of these plots is included in the Supplementary Materials to compare the effects of CA in each situation of hold or polish. The values of *E*_corr_ and *i*_corr_ obtained from the polarization plots in Figure 8 for Mo are presented in Figure 10 panels A–D, while the corresponding parameters for the Cu sample are shown in panels E–H of the same figure. The *E*_corr_ values for Mo and Cu are also compared with their corresponding values of *E*_OC_. These *E*_OC_ values for the (H) and (P) cases have been used to generate the plots in Figure 6B and Figure 7B, respectively. 

As seen in Figure 10C,D, the values of both *E*_OC_ and *E*_corr_ follow the same trend with respect to variations in [CA] and [SiO_2_], although the absolute values of the two potentials are different due to their different measurement conditions [12,66]. According to Equations (5) and (6), the surface makeup of the Mo sample in the CA-free slurries is dominated by (MoO_3_ and) MoO_3_·2H_2_O, which can act as anodic as well as cathodic inhibitors for Mo [39]. Under stationary hold, surface films of these oxides attain their stable configurations and fully manifest their function as reaction inhibitors. Thus, both the cathodic and anodic branches of the polarization plots (H) in Figure 8A–C are shifted toward smaller currents with respect to their accompanying plots (P). 

The faradaic activity of Mo mostly returns to its intrinsic level as these films are removed by mechanical abrasion. A comparison of the relative placements of the anodic and cathodic current branches on the horizontal axis shows that anodic suppression of the currents by MoO_3_/MoO_3_·2H_2_O in the H case is stronger than the corresponding cathodic passivation [*X*_a_ < *X*_c_ in Equation (18)]. For this reason, and since Equation (18) for *E*_OC_ also applies to *E*_corr_, Figure 10C,D shows that *E*_corr_ (H) > *E*_corr_ (P); the same behavior of *E*_OC_ (H) and *E*_OC_ (P) has already been discussed in the context of Figure 6. 

In the cases of Figure 8D–F, the Mo surface represented by plots (H) is expected to be mostly covered by the Mo-citrate complex, MoH_−1_Cit(OH)_2_, considered in Equation (8); the Mo surface represented by the accompanying plots (P) should be essentially free of this complex and Mo-oxides. The Mo surface containing adsorbed Cit^3−^ species appears to exhibit selective characteristics of a cathodic promoter in the presence of SiO_2_ on the sample surface. This can be seen by noting that the currents in the anodic branches of plots (H) and (P) in Figure 8D–F are mutually comparable to a large extent, while the currents in the corresponding cathodic branches of the plots (H) (especially in panels E and F) are measurably shifted toward higher currents compared to those of their accompanying plots (P). 

It is likely that under stationary hold, co-adsorptions of SiO_2_ particles and Cit^3−^ in the Mo surface stabilize the abrasives; the SiO_2_-containing surface sites then serve to facilitate subsequent adsorption and reduction of H_2_O_2_ at those sites [67] and contribute in this way to the Mo sample’s overall cathodic activity. As a net result, the CA containing slurries for Mo correspond to a case where *X*_c_ > *X*_a_, so that *E*_corr_ (H) and *E*_OC_ (H) have higher values than *E*_corr_ (P) and *E*_OC_ (P), respectively (Figure 6B and Figure 10C,D). 

In the description of Equations (11) and (12), the Cu test surface under stationary hold in the absence of CA mainly contains Cu_2_O and Cu(OH)_2_ species. A potentiodynamic scan in the direction of increasing voltages in this case increases the hydroxide coverage of the Cu surface by activating the anodic reaction [43]:

Cu_2_O + H_2_O + 2OH^−^ = 2Cu(OH)_2_ + 2e^−^ (19)

and at least part of the Cu(OH)_2_ produced in this way chemically converts to CuO as noted below Equation (12). In the presence of surface abrasion, the Cu_2_O sites needed for reaction (19) are removed from the Cu surface along with the other surface species of Cu(OH)_2_ and CuO. Thus, when Cu_2_O is present on the Cu surface represented by the (H) plots in Figure 9A–C, reaction (19) is responsible for the higher anodic currents shown by these plots compared to those shown by their accompanying plots (P). The presence of Cu_2_O on the Cu surface does not have significant effects on the cathodic currents of the plots in Figure 9A–C, since this oxide can support certain cathodic activities of Cu, including the ORR [45,68]. Due to this reason, the Cu surfaces tested in Figure 9A–C favor the condition, *X*_a_ > *X*_c_ ≈ 1, which is also manifested in the data of Figure 7. 

A comparison of plots (H) and (P) in Figure 9D–F does not reveal any clearly identifiable anodic signatures of Cu_2_O-supported reaction (19) in the stationary hold situation. Accordingly, it is evident that the Cu_2_O component of the surface film of Cu is mostly eliminated due to this oxide’s conversion to (CuCit_2_H_−2_)^4−^, as indicated in reaction (13). In alkaline media, reaction (4) usually occurs via an intermediate, deprotonated species of hydrogen peroxide, HO_2_^−^ (hydroperoxyl anion) [69]. At the same time, under stationary hold of the Cu sample in the CA containing slurries, the Cu surface generates a sizable amount of (CuCit_2_H_−2_)^4−^ anions within the sample’s interfacial liquid. By Coulombic repulsion, these anions of strong negative charge interact with the HO_2_^−^ anions to suppress the cathodic currents, and the results are detected as plots H in Figure 9D–F. This effect of cathodic suppression grows stronger in the presence of SiO_2_, most likely by interactions between (CuCit_2_H_−2_)^4−^ and silica. Interactions of this type are expected in the present situation by considering the known interactions of citrate with SiO_2_ in aqueous media [70]. 

The oxide/hydroxide layers of Cu and interfacial accumulations of (CuCit_2_H_−2_)^4−^ are effectively removed under surface abrasion of Cu. Consequently, the cathodic current branches of the plots (P) in Figure 9D–F acquire higher currents than those of the corresponding plots (H). Thus, the data in the right column of Figure 9 represents the situation *X*_a_ < *X*_c_ in Equation (18); consequently, the values of *E*_corr_ (H) and *E*_OC_ (H) in these cases appear below their corresponding values detected under polish. 

The corrosion current densities (*i*_corr_) for the metal test samples were determined by Tafel extrapolations of the potentiodynamic polarization plots in Figure 8 and Figure 9. These *i*_corr_ data are shown in Figure 10A,B [for Mo], (E) and (F) [for Cu]. The overall values of *i*_corr_ (H) measured in this work are comparable to those reported by other authors for Mo [7,17] and Cu [30,71] using stationary samples in alkaline polishing slurries of similar makeups (based on moderate concentrations of oxidizers, complexing agents, and abrasive particles). The present results indicate how the values of *i*_corr_ (P) increase with respect to those of *i*_corr_ (H) due to the activation of tribo-corrosion in the polish case. 

Although corrosion currents in their traditional definition generally imply the equilibrium currents of metal dissolution (coupled with some oxidant reduction), as noted in the discussion of Equation (1), corrosion currents linked to metal CMP cover a broader range of anodic reactions [such as Equations (5), (6), (9), (10) and (13)] that are responsible for electrochemically reducing the metal’s surface hardness. Referring to the discussions of Figure 4 and Figure 6, the *i*_corr_ values plotted in Figure 10 for Mo are chiefly dictated by the mixed reaction (7) with indirect effects of the chemical reaction (8). Likewise, according to Figure 5 and Figure 7, the *i*_corr_ data in Figure 10C are linked to reactions (9)–(15) for Cu. To discuss the implications of the corrosion currents for CMP, in the next section, the measured values of *i*_corr_ are converted to those of their corresponding corrosion rates (CRs) and tribo-corrosion rates (TCRs). 

### 3.9. Comparison of Tribo-Corrosion Rates and Material Removal Rates

**Figure 11:**
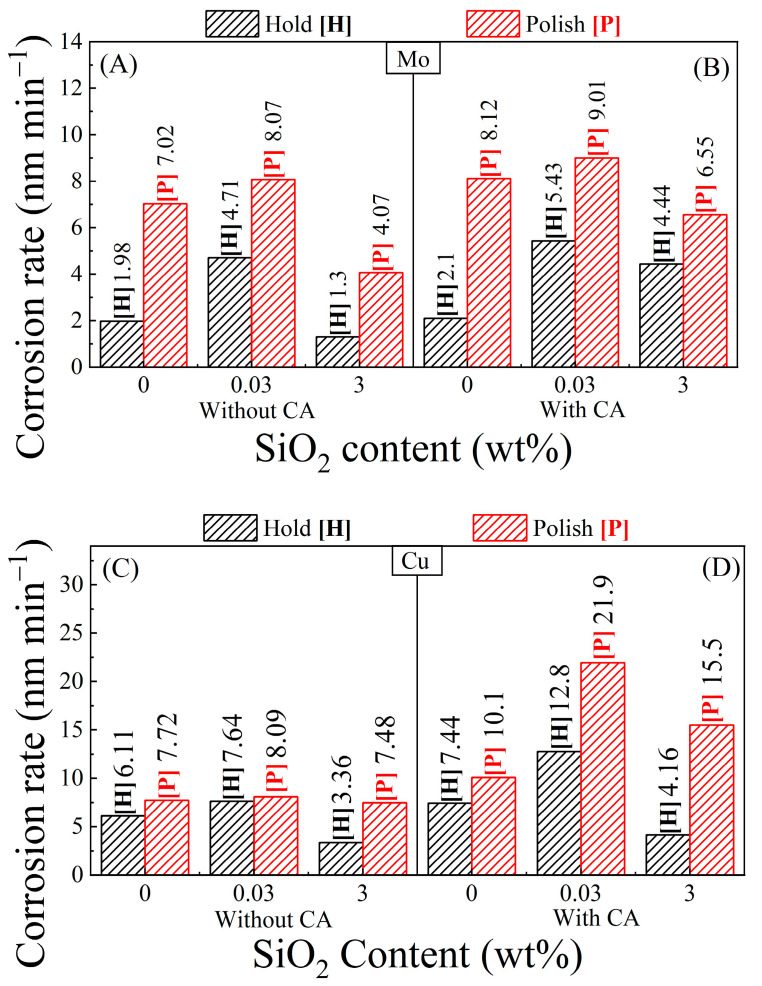
Corrosion rates of Mo [(**A**,**B**)] and Cu [(**C**,**D**)] samples compared using different slurry compositions based on SPC, CA and SiO_2_. These results were obtained from the polish [P] vs. hold [H] corrosion current data in Figure 10. The number associated with each bar denotes the value of the variable represented by that bar.

Figure 11 shows the CR values for Mo (A and B) and Cu (C and D) calculated from the corresponding *i*_corr_ data in Figure 10 according to the formula: *CR* = *M* *i*_corr_/*ρzF*. Here M and *ρ* denote, respectively, the molecular weight and mass density of the material being planarized. SiO_2_ particles adsorbed at the CMP metal’s surface serve as efficient adsorption sites of H_2_O_2_. The primary stage of this process involves molecular adsorption [67], which can be linked to the known property of SiO_2_ for stabilizing H_2_O_2_ [47]. The inclusion of 0.03 wt% SiO_2_ in the slurry provides stable adsorption sites of H_2_O_2_ at the CMP interface and consequently promotes the subsequent electroreduction of H_2_O_2_ described in Equation (4). Since this reaction directly contributes to metal corrosion [via those of reactions (7) for Mo and (11) as well as (12) for Cu], the *CR*(*H*) values increase correspondingly. 

At 3 wt% SiO_2_ content of the slurry, the increased surface coverages of adsorbed SiO_2_ on Mo and Cu provide additional abrasive sites for slow dissociative adsorption of H_2_O_2_, following an initial step of molecular adsorption [67]. Competing with reaction (4), such a process can correspondingly decrease the metals’ *CR*(*H*). Moreover, since the isoelectric point of SiO_2_ exists at around pH = 2 [72], the abrasive particles adsorbed onto Mo and Cu at pH 8 contain negative charges. Coulombic repulsion of these charged abrasives can hinder the underlying metal’s adsorption of OH^−^ that is necessary to sustain reactions (5), (6), (9) and (10). At high surface coverages of SiO_2_, such an effect can compete against the corrosion-supporting feature of the abrasives that comes from the latter’s affinity to adsorb H_2_O_2_. This electrostatic effect can also contribute to the lowering of *CR*(*H*) values observed in Figure 11 in the transition from 0.03 wt% to 3 wt% SiO_2_ containing slurries. The *CR*(*P*) values of Mo and Cu mostly maintain the [SiO_2_]-dependent trend of *CR*(*H*). 

**Figure 12:**
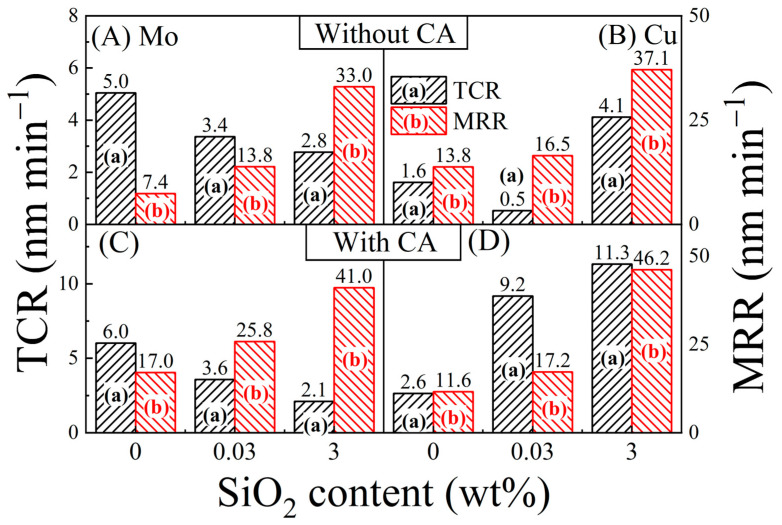
(a) Tribo-corrosion rates (scale on left vertical axis) and (b) material removal rates (scale on right vertical axis) of Mo [(**A**,**C**)] and Cu [(**B**,**D**)] samples compared using different slurry compositions based on SPC, CA and SiO_2_. These results were obtained from the polish- vs.-hold corrosion current data presented in Figure 10. The CA contents of the slurries are 0.0 M in [(**A**,**B**)] and 0.1 M in [(**C**,**D**)]. The number associated with each bar denotes the value of the variable represented by that bar.

*TCR* values are determined using the definition [60]: *TCR* = [*CR* (*P*) − *CR* (*H*)], where the *CRs* for the polish and hold cases are taken from Figure 11. In a phenomenological approach, the *TCR* can be considered equivalent to the term *RR*_wc_ (within a factor of proportionality) in Equation (1). Figure 12 compares the MRRs (from Figure 2) for Mo and Cu in the different test slurries with their corresponding TCRs. To bring out the main features of the data, different optimized scales have been used for the two quantities plotted in Figure 12. The following two main observations can be made from the data compiled in Figure 11 and Figure 12: (i) the MRRs are considerably higher than their corresponding CRs and TCRs for all the CMP systems examined here; (ii) the extent and the nature of the slurry-dependent MRR trends are different for Mo and Cu. The mechanisms of these effects are examined below. 

MRRs in metal CMP almost always exceed the corresponding electrochemically measured CRs [28,73] and TCRs [66]. To account for this phenomenon in metal CMP, it has been suggested that a material removal mechanism operates in addition to the (corrosion-like) mechanism of surface complex formation [31]. This additional mechanism of material (wear and) removal can be linked to the mechanical action of abrasion that extends into the metal underlying the chemically modified surface region [32]. The rate of this process can be phenomenologically associated with the rate of corrosion-induced wear, *RR*_cw_, in Equation (1) [26,30]. According to recently available CMP data, the removable material contributed by this process likely comes from structural destabilization of metal layers adjacent to the chemically modified (passivated/corroded) surface region [32]**.** Choi et al. have proposed a similar mechanism of copper CMP, which is based on mechanical removal of (un-complexed) Cu from structurally destabilized regions of selective plastic deformation where crystallographic defects are formed in the metal [28].

Tribo-corrosion models of metal CMP typically propose that material wear for removal is initiated by corrosion-like formation (and mechanical removal) of complexed or oxidized surface films. The MRRs supported by this process correspond to the electrochemically measured values of TCRs. At the same time, strain mismatch between the oxidized metal layer and its underlying metal forms ductile or brittle fractures at the oxide–metal and/or complex-metal boundaries. Removal of material affected by this type of wear can be attributed to the term *R*_cw_ included in Equation (2).

The component *R*_cw_ of material removal from the zones affected (with mechanical wear or fracture) by their neighboring corroded regions is not accounted for by the electrochemically measured *CR*s and *TCRs*. In agreement with previous results [60,66,74], a correlation is observed here between the TCRs and MRRs for the Cu CMP system in Figure 12D, and to some extent for the case of Mo in Figure 12C. This correlation is generally expected if the MRRs due to strain-mismatched wear are proportional to the TCRs. On the other hand, if the frictional energy of CMP is not proportionately consumed between the processes of material removal from a metal’s chemically modified and unmodified regions, the net MRR does not generally exhibit a clear correlation with the TCR. The Mo CMP systems considered in Figure 12A,B correspond to this latter case. 

While surface chemistry largely dictates the material selectivity of CMP, the role of this chemistry in material removal from lattice-mismatched deeper layers of a metal is relatively indirect. As a result, depending on the system, the trend of CMP selectivity observed at low abrasive concentrations may cease to operate at higher abrasive concentrations, although the MRR may continue its increasing trend with increasing abrasive concentrations. This latter scenario can be associated with the different trends found between the CMP selectivity and the absolute MRRs of Cu and Mo in Figure 2 and Figure 3.

## 4. Conclusions

The experiments reported here indicate how the CMP selectivity between Cu and its barrier metal can be varied by utilizing the different surface chemistries of the metals in strategically designed polishing slurries based on adjustable amounts of an oxidizer, a complexing agent, and abrasive particles. Inclusion of 3 wt% SiO_2_ abrasives to a base slurry of [0.1 M KNO_3_ + 20 mM SPC at pH = 8.0] yields a 4.5-fold increase in the value of MRR (Mo), while the MRR (Cu) correspondingly increases by a factor of 2.7. Using a CA-based slurry solution of [0.1 M KNO_3_ + 20 mM SPC + 0.1 M CA], these metal-specific effects of SiO_2_ are nearly reversed, where the values of MRR (Mo) and MRR (Cu) are augmented by 2.4 times and 4.0 times, respectively, from their corresponding values measured with the abrasive-free formulation. Based on these results, it is found that a slurry composition of [0.1 M KNO_3_ + 20 mM SPC + 0.1 M CA + 3 wt% SiO_2_] can maintain the [Mo:Cu] CMP selectivity at a value of 0.89, where both the Mo and Cu sample surfaces are planarized at closely comparable rates of 46 and 41 nm min^−1^, respectively. By lowering (to 0.03 wt% [SiO_2_]) or eliminating the abrasive content of this CA-based formulation, the [Mo:Cu] CMP selectivity can be increased to ~1.5 with some loss of the absolute MRRs. 

The above observations are clarified in terms of competitive actions of the chemical and mechanical components of CMP. In slurries containing zero or low concentrations of SiO_2_, the chemical component of CMP dominates where chemically dictated selectivity of MRRs operates. At higher SiO_2_ concentrations, the abrasive action of these particles further promotes the mechanical component of CMP, which then governs the MRRs for both Mo and Cu by overriding the chemical criteria of CMP selectivity between the two metals. The MRR selectivity values measured under these conditions do not correlate with those of CRs and TCRs, as the abrasive enhanced component of material removal remains undetected in the electrochemically measured values of CRs/TCRs. 

The present experimental results also indicate the metal-specific mechanisms of material removal from Mo and Cu in the experimental slurries. In the citrate-free weakly alkaline slurries, H_2_O_2_ released from SPC oxidizes the Mo surface to MoO_3_·2H_2_O, which serves as the main removable material for CMP. In citrate-containing slurries, surface films of a Mo-citrate complex, most likely with a makeup of MoH_−1_Cit(OH)_2_, act as the removable material for CMP. On the Cu sample surface, Cu-hydroxide/oxide species serve as the main removable material in the citrate-free slurries tested here. When present in the slurry, citrate ions moderately increase the MRR of the Cu sample by structurally weakening surface species of Cu-hydroxide/oxide through the formation of soluble Cu-citrate complex anions within the oxidized surface layers of Cu. 

While the findings of this study are centered on a specific set of test systems involving Mo and Cu CMP, the considerations explored here for comparing the strengths and selectivity of MRRs should apply to CMP systems of other barrier materials and Cu. For instance, the tribo-electrochemical investigative approach of this study has also been demonstrated for other CMP systems based on ruthenium–copper [74], tantalum–copper [64], and cobalt–copper [32,66] combinations. Moreover, the detailed tribo-electroanalytical protocols demonstrated in this work for probing the mechanisms of material selective CMP are not system specific and can be readily extended to study other similar CMP systems. Such experiments aimed at simultaneously mimicking the chemical and mechanical conditions of CMP in a laboratory setting can serve as a relatively cost-effective tool to aid the development of advanced CMP slurries for back end of the line processing in IC manufacturing.

## Figures and Tables

**Table 1:** Chemical compositions and ohmic resistances of test slurries.

Slurry	Composition	*R*_S_ (Ω) (Mo, Cu) ^(a)^
a	0.1 M KNO_3_ + 20 mM SPC (Ref)	47.1, 32.7
b	Ref + 0.03 wt% SiO_2_	48.0, 48.6
c	Ref + 3 wt% SiO_2_	52.4, 55.2
d	Ref + 0.1 M CA	15.3, 11.5
e	Ref + 0.1 M CA + 0.03 wt% SiO_2_	29.7, 19.7
f	Ref + 0.1 M CA + 3 wt% SiO_2_	33.7, 23.7

^(a)^ Note: The first and second entries in each row of the last column represent the Mo and Cu samples, respectively. Formulation (a) is the reference (Ref) slurry, which forms the base solution for all six test slurries.

**Table 2:** Impedance variables of the circuit model in Figure 4B for Mo.

Parameter	Slurry					
	a	b	c	d	e	f
*R*_sc_ (Ω cm^2^)	238.6	243.5	369.3	78.2	153.8	174.8
*Y*_d_ (μS s^d^ cm^−2^)	385.3	335.3	748.1	252.6	228.4	182.2
*d*	0.79	0.79	0.74	1	1	1
*R*_p1_ (Ω cm^2^)	68.0	59.2	390.4	27.6	79.9	86.6
*R*_p2_ (Ω cm^2^)	ND ^(b)^	ND	ND	93.8	242.8	280.9
*σ*_a_ (Ω cm^2^ s^−0.5^)	79.4	72.7	50.9	ND	ND	ND
*C*_a_ (μF cm^−2^)	ND	ND	ND	405	354	283

^(b)^ ND denotes not detected.

**Table 3:** Impedance variables of the circuit model in Figure 5B for Mo.

Parameter	Slurry					
	a	b	c	d	e	f
*R*_sc_ (Ω cm^2^)	258.8	385.0	437.5	91.3	156.3	187.6
*Y*_d_ (μS s^d^ cm^−2^)	2.50	1.69	0.97	48.8	101.5	55.8
*d*	1	1	1	0.77	0.63	0.74
*R*_a_ (Ω cm^2^)	55.5	128.7	60.5	58.0	515.1	106.2
*Y*_a_ (μS s^a^ cm^−2^)	22.5	22.9	12.7	3.57	2.54	5.19
*a*	0.83	0.79	0.85	1	1	1
*R*_p_ (Ω cm^2^)	1065	2387	5215	195	232	236

## Data Availability

Data are contained within the article.

## Supplementary Materials

The following supporting information can be downloaded at: https://www.mdpi.com/article/10.3390/ma17194905/s1, Figure S1: Speciation of citric acid as a function of slurry pH; Figure S2: (A) Schematic of a pad-sample interface, and (B) a general electri-equivalent circuit (EEC) describing the CMP interface. (C) A simplified version of the EEC from B for large values of the capacitance, CI, RE and WE (CMP sample) denote the reference electrode and the working electrode, respectively; Figure S3: Effects of CA on (iRs corrected) polarization plots for a molybdenum sample examined under polishing in weakly alkaline CMP slurries. The concentrations of different additives in the slurries are indicated in the figure; Figure S4: Effects of CA on (iRs corrected) polarization plots for a copper sample examined under polishing in weakly alkaline CMP slurries. The concentrations of different additives in the slurries are indicated in the figure; Table S1: Fitting Errors for Impedance Parameters Obtained by CNLS Fitting EIS Data in Figure 4A for Molybdenum; Table S2: Fitting Errors for Impedance Parameters Obtained by CNLS Fitting EIS Data in Figure 5A for Copper.
