# Supplementary material for: Mechanisms of Chemically Promoted Material Removal Examined for Molybdenum and Copper CMP in Weakly Alkaline Citrate-Based Slurries
Source: Materials (Basel). 2024 Oct 7;17(19):4905. doi: 10.3390/ma17194905 (PMC11477894; doi:10.3390/ma17194905)
Supplement: Supplementary file 1 [file materials-17-04905-s001.zip › materials-3182040-supplementary.pdf]

Supplementary Materials

Mechanisms of Chemically Promoted Material Removal Examined for Molybdenum and Copper CMP in Weakly Alkaline Citrate Based Slurries

K. U. Gamagedara and D. Roy *

Department of Physics, Clarkson University, Potsdam, NY 13699-5820, USA

S.1. Speciation of Citric Acid as a Function of Solution pH

The figures, tables, and equations included in this Supplementary Section are referred to as Figure S.1, Figure S.2, Table S., Equation S.1, etc. for the purpose of differentiating them from those of the figures, tables and equations in the main article.

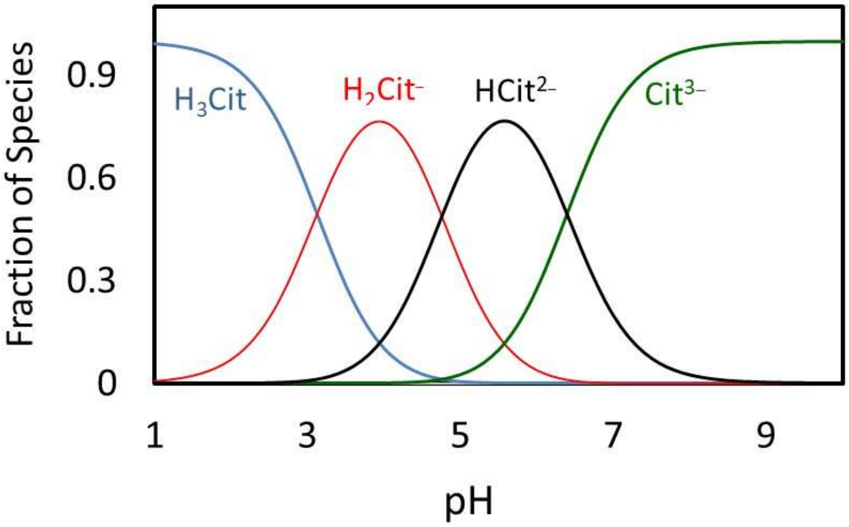

Figure S.1. Speciation of citric acid as a function of slurry pH.

Figure S.1 shows calculated distribution of pH dependent citric acid ($C_6H_8O_7$) species. Here, $C_6H_8O_7 = H_3Cit$, with $Cit = C_6H_5O_7$. As shown in the diagram, the predominant species of citric acid (CA) is Cit^{-3} at pH = 8 used for the experimental slurries in the present work.

S.2. CMP Strategy and Considerations for Slurry Selection

In metal CMP, it is customary to chemically form a thin, structurally weak film of a surface complex/oxide that can be readily removed by low down-pressure (low-P) abrasion. This CMP strategy of surface modification is based on the considerations of Preston's and Archard's laws, according to which the material removal rate (MRR) of CMP is expressed as [1]:

$$MRR = K_P PV = \frac{K_a}{H_s} PV \tag{S.1}$$

where K_P ($\equiv K_a/H_s$) is the Preston coefficient; V is the relative velocity between the pad and the surface being polished. K_a is the dimensionless Archard wear coefficient, and H_s is the mechanical hardness of the modified surface layer. For CMP, the value of H_s is intended to be considerably lower than that of the metal being processed.

While several modifications of Preston's law are found in the literature, the traditional form of Equation (S.1) is useful to note the basis for chemically modifying the CMP material's surface layer. Since K_a is a constant of the polishing mechanism and V is typically operated within a moderate range, chemically reducing the CMP material's hardness can yield high values of MRR while maintaining the polishing pressure, P, in Equation (S.1) at a relatively low value. Low-P settings are necessary in CMP to avoid damages to delicate IC features like the dielectric materials underlying metal components. The CMP metal's modified surface layer generally is composed of the metal's oxides and/or complexes. Planarization of the CMP surface occurs through continuous mechanical removal and (electro)chemical regeneration of these oxides/complexes.

Usually the CMP supporting surface complexes (formed by selected complexing agents in the polishing slurry) are preferred to be insoluble or weakly soluble to avoid metal dishing from excessive dissolution of the surface layers. In the absence of metal ions generated by dissolution, the complex forming reactions tend to be better facilitated by oxidized forms of the metal than the metal itself. Due to this reason, an intermediate step of surface oxidation is commonly operated by oxidizer additives in the slurry to generate surface complexes in metal CMP. If not entirely consumed for complex generation, the oxide species remain mixed with metal complex(es) at the metal's modified surface. To support the essential functions of CMP, the polishing slurry contains at least an oxidizer, a complexing agent, and abrasive particles, along with a pH adjuster to utilize the metal's pH specific surface chemistry. Inclusion of a dissolution inhibitor in the slurry becomes necessary if the CMP surface exhibits strong dissolution. Dissolution inhibitors are excluded from the present slurries to explore the option of inhibitor-free CMP, a method often considered for alleviating CMP defects [2].

Reducing excessive dissolution of Mo is an essential criterion of Mo CMP [3], and in acidic slurries containing H_2O_2, CMP surfaces of Mo tend to generate a soluble species, MoO_4^- [4] . Hence, to minimize strong surface dissolution of Mo, the acidic pH range is avoided for the present test slurries. At the same time, in the strong alkaline pH environment, colloidal silica abrasives in some H_2O_2 based slurries have been found to interact with Mo oxide surface sites to suppress removal rates of Mo [5]. Although other alkaline slurries containing H_2O_2 and silica have not shown this effect, abrasive-free CMP of Mo in alkaline slurries have also been employed previously to avoid possible inhibiting effects of abrasives. In view of these earlier findings, a weakly alkaline pH setting is used here with colloidal silica concentrations varied between 0 and 3 wt%, to check the effects of abrasives on Mo CMP.

It is useful to note here that the standard potential (E^0) for electro-dissolution of Mo ($Mo = Mo^{3+} + 3e^-$) is −0.44 V vs. SCE. This value is notably lower than the value, 0.34 V vs. SCE, of E^0 reported for Cu electro-dissolution ($Cu = Cu^{2+} + 2e^-$) [6]. Comparing these relative standard potentials alone, Mo is expected to corrode more easily than Cu when both metals are combined. However, standard potentials do not account for (non-standard conditions arising from) the effects of reactant concentrations and additional redox reactions supported by the slurry-additives. These latter effects are included in the metal specific Nernst potentials calculated using the Nernst equation, and the results can be assembled in Pourbaix (potential-pH) diagrams. For this reason, Pourbaix diagrams are commonly used as standard guidelines for designing the initial chemical compositions of

CMP test slurries. This latter approach has been used in this work to select the slurry chemicals.

As noted in Equations (2)-(15), surface modification of a metal for CMP occurs through multiple reactions, and the OCP of the system results as an equilibrium mixed potential of these reactions. Depending on the pH and other details of a given slurry, direct electro-dissolution steps (like those of Mo/Mo^{3+} and Cu/Cu^{2+}) may not play active roles in determining this OCP. According to the published Pourbaix diagrams of Mo, thermodynamic stability of electro-dissolved Mo^{3+} in aqueous solutions is expected in acidic solutions, at pH <3 [4]. Likewise, for the Cu CMP system, the electro-dissolution process of generating thermodynamically stable Cu^{2+} is generally favored in acidic solutions [7]. On the basis of these considerations, the Mo/ Mo^{3+} and Cu/Cu^{2+} dissolution reactions have not been considered as major contributors to the CMP reactions of Mo and Cu, that would be expected to operate at the alkaline slurry pH used here.

A close inspection of the data in Figures 6 and 7 indicates that the OCP-differences [(E_{OC}-Cu)−(E_{OC}-Mo)], measured between Mo and Cu are small compared to the difference of standard potentials for these metals' electro-dissolution (~0.25 and 0.4 fractions of E^0 under the hold and polish conditions, respectively). As noted in the discussions of these figures, the oxide/complex layers developed on a metal surface in a CMP slurry serve as removable materials under abrasion, and tend to chemically passivate the CMP metal's surface. The different surface coverages of these species established in the hold and polish situations differently affect the values of ($i_{rc}A_c/i_{ra}A_a$) in Equation (17), and the OCP values are affected accordingly. In the slurries used here without external corrosion inhibitors, the products of reactions (5)-(7) represent the passivating surface species of Mo and those generated via reactions (9)-(12) act to passivate Cu.

By using selective corrosion inhibitors, the Cu-barrier OCP differences can be further reduced to minimize the likelihood of corroding Mo in its bimetallic contact with Cu in a CMP situation. Choosing such inhibitors is a relatively nontrivial task, since it is difficult to utilize the faradaic (anodic vs. cathodic) selectivity of these inhibitors in a metal-specific way *under surface abrasion* for CMP applications. Furthermore, the corrosion variables, as well as the MRRs of a metal film in CMP would vary depending on the grain characteristics and crystalline structure of the film, as dictated by the details of the film deposition methods. These characteristic features of metal films introduce additional complexities to the protocols for setting up corrosion-control schemes involving bimetallic CMP systems. These issues constitute an independent topic for investigation, and will be discussed further in a separate report.

S.3. Rates of Surface Corrosion and Material Removal

Interpretations and phenomenological treatments of the different terms in Equation (1) somewhat vary in the literature depending on the specific models employed to analyze MRR data. In tribo-electrochemical analyses of CMP mechanisms, the RR_c term in Equation (1) can be taken as: $RR_c \approx$ SER + RR_{cf}, where RR_{cf} is the rate of material removal from the CMP enabling surface films that are generated by corrosion-like (oxidation and complex-forming) reactions. Similarly, $RR_{wc} \approx DER + RR_{wcf}$, where DER is the dynamic etch rate established under surface abrasion, and RR_{wcf} is the rate of material removal from the additional component of surface films generated due to mechano-chemically induced reactions under mechanical abrasion. RR_{cw} is typically dictated by the rate of wear generation in the metal underlying the surface regions affected by corrosion. CMP (electro)chemically generates these 'soft' surface films for mechanical removal under low down-pressure. This mechanism applies to the terms RR_{cf}, RR_{wcf}, and RR_{cw} that are embedded in Equation (2).

S.4. Additional Details of Materials and Methods

The core components of Materials and Methods have been presented in the main article; some relevant additional details of these topics are discussed below. The Mo disc sample's (Kurt Lesker Part Number EJTMOXX351A4), Mo content was 99.95%, with small traces of Al, Si, Mg, C, and Ca. The Cu disc (Part Number EJTCUXX401A4) was 99.99% pure with negligible traces of Ag, Bi, Fe, Mn, Ni, and other elements.

Reagent grade chemicals obtained from Fisher Scientific were employed with triple distilled water to prepare slurry solutions. The KNO_3 component of the slurry was necessary to promote the ionic conduction needed for electrochemical measurements. SPC served as a cost effective and environmentally compatible solid carrier of H_2O_2, an essential oxidizer for metal CMP. The surface of each metal sample was prepared in several steps before each experiment, starting with repeated rinsing in distilled water and polishing with gradually finer grits of sandpapers. The sample was then polished in steps with aqueous slurries of 1.0, 0.5 and 0.05 µm alumina powder using a locally built rotary polisher of 7.62 cm diameter platen, equipped with a pad of Buhler Microcloth. Before every data-run, the sample was thoroughly rinsed in triple distilled water. After its insertion in the experimental cell, the open circuit potential (OCP, or E_{OC}) of the sample interface was allowed to stabilize for 10 min before starting data collections.

For the test cell, the primary mechanical controls of CMP included the down-pressure of polishing, along with independent adjustments of the rotation speeds of the sample and the platen-pad assembly. Electrical connections to the moving WE were made with copper rings that were in contact with carbon brushes connected to shielded coaxial cables leading to the potentiostat. Intermittent OCP transients were recorded while alternately applying 4 min long sequences of H and P conditions at the sample-pad interface. For the potentiodynamic measurements, previously described procedures were used to ensure that the chosen scan speed of LSV was suitable for the experimental systems examined [8].

Complex nonlinear least square (CNLS) analyses of the EIS data were carried out using Zsimpwin Software. CNLS fits were accepted if they yielded low (typically $\leq$ 10%) uncertainties in all the calculated EEC elements. Aside from checking the surface reaction characteristics, EIS was used to determine the ionic conductivity of each test slurry, as well as the ohmic resistance (R_s) of the slurry in the test cell's working electrode-reference electrode (WE-RE) gap. These ex-situ conductivity measurements for the slurries were performed using a Teflon cell equipped with two identical stainless-steel electrodes.

Since the polishing platen and the pad were attached to the test-cell's base, the entire lower part of the cell was rotated (along with the sample holder situated in the cell's upper part). As described elsewhere in detail [9], the accommodation of this rotating cell base required a RE compartment separated from the main cell's slurry reservoir. This isolation of the SCE reference was necessary to avoid possible seepage of Cl^- ions from the RE into the slurry (as Cl^- could potentially serve as a surface pitting reactant, especially for Cu). The RE was electrically (ionically) connected to the main slurry via a C-shaped salt bridge (SB) of 33.6 cm total length, whose resistance was determined in separate EIS experiments. Including this SB arrangement, the WE was separated by a slurry-path of 38 cm, which resulted in the R_s values listed in Table 1.

These R_s data were collected in a non-contact mode by leaving a gap of ~1 mm between the sample surface and the polishing pad surface with both fully immersed in the slurry. This arrangement was necessary to avoid altering the values of R_s due to an interfacial resistance (R_I) contributed by pad asperities and abrasives in the pad-sample contact mode. This resistance, R_I, appeared in the net slurry resistance (R_{sc}) measured in the contact mode. The different implications of the latter resistances are elaborated in the next section of Supplementary Material. The values of R_s from Table 1 were used to determine ohmic corrections to the LSV data. The necessity and the protocols used for these ohmic corrections have been described in a previous publication [9]. The data for OCP transients and LSV were processed using Origin software. The *Tafel Extrapolation App* of Origin was

used to determine corrosion potentials and corrosion currents from potentiodynamic polarization data.

S.5. Slurry Resistances Measured in Non-Contact and Contact Modes of Pad-Sample Combination

Figure S.2A shows a commonly considered schematic (not drawn to scale) of the pad-sample interface for metal CMP where the sample is arranged in a contact mode with the pad surface. This drawing includes the metal surface being polished, the pad asperities, and (active as well as inactive) abrasives. The slurry fluid at a stationary sample interface resides in the pockets formed by pad asperities. During polishing, the interfacial slurry forms a fluid film of ~20 µm thickness. The width of the multi-component interfacial region can be taken as the average height of pad asperities (which depends on the down-pressure of CMP), and is indicated in the figure as δ.

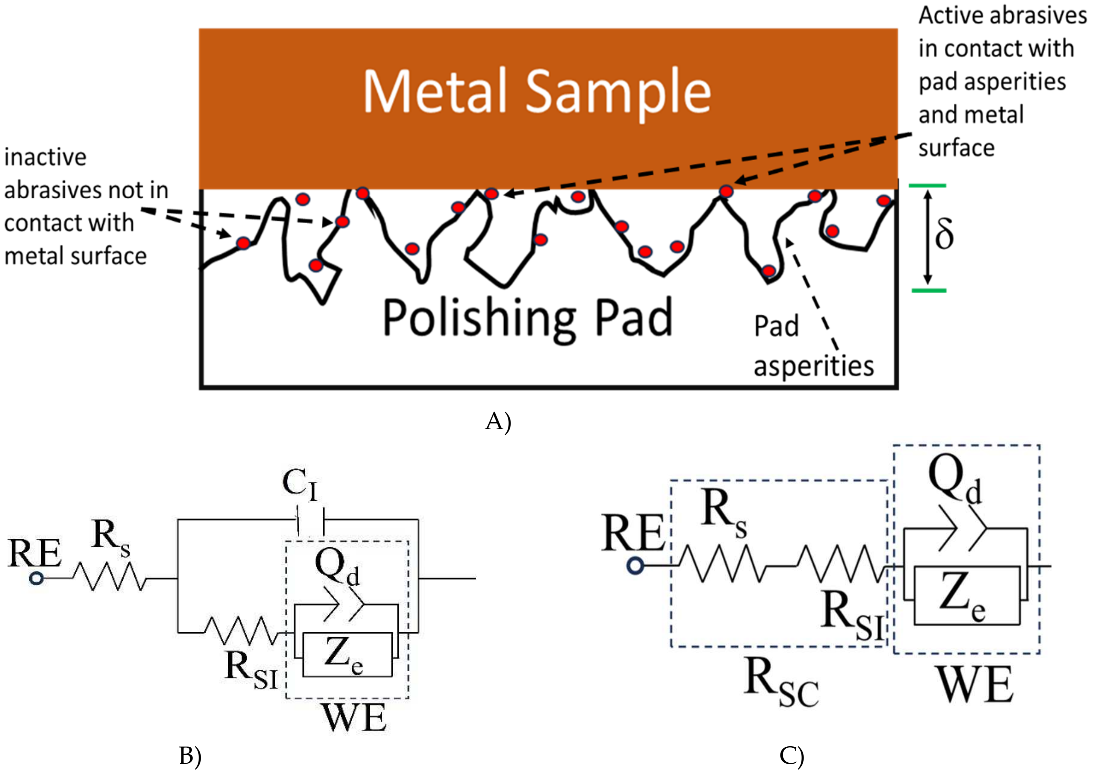

Figure S.2. (A) Schematic of a pad-sample interface, and (B) a general electri- equivalent circuit (EEC) describing the CMP interface. (C) A simplified version of the EEC from B for large values of the capacitance, C_I. RE and WE (CMP sample) denote the reference electrode and the working electrode, respectively.

Panel B in Figure S.2 displays a general EEC used to describe the impedance characteristics of the interface shown in A. Traditionally, this EEC has been used to characterize porous surface coatings on metals; the adaptation of this EEC for the system in Figure S.2.A is based on the similarities between the physical configurations of the pad-sample interface and the corrosion protective surface layers with perforations.

R_s in Figure S2.A is the ohmic resistance of the slurry (solution) residing between the WE and RE. This R_s can be measured in the non-contact mode of sample setting, where the sample surface and the pad surface are placed at ~1cm separation in a mutually parallel configuration, both being fully immersed in the experimental slurry. For an electrochemically probed CMP interface, C_I and R_I denote, respectively, the capacitance and the

resistance of the interface region containing the slurry solution, abrasives, and pad asperities. The complex impedance of the WE is represented by a general element, Z_e, the detailed composition of which is system specific. Q_d is the usual double layer CPE. Since the pad asperities and silica particles (both electrically non-conducting) occupy a significant fraction of the interface layer, it is found that $R_I > R_s$ in most cases, which is expected in view of the effective medium theories of electrical resistivity.

The area-normalized value of C_I can be approximated as: $C_I \approx (\varepsilon_{eff} / \delta)$, where ε_{eff} is the effective dielectric function of the (multi-material) interface region. For low abrasive concentrations, the value of ε_{eff} is determined primarily by those of the polyurethane pad and the slurry. Since the characteristic height of pad asperities (~δ) under the typical conditions of CMP is at least three orders of magnitude larger than the electrochemical double layer thickness, the admittance ($j\omega C_I$) of C_I tends to be small compared to that of the (R_I-Z_e) combination connected in parallel with C_I. Here, ω is the perturbation frequency of EIS, and $j = (-1)^{1/2}$. In these cases, C_I remains undetected in the CNLS analyses of EIS data, and consequently, calculated fits to the EIS data typically lead to the EEC shown in Figure S2.C, which is a compact version of the model in Figure S.2.B. The two ohmic resistances in Figure S.2.C is detected as an effective slurry resistance, $R_{sc,}$ in the metal sample's contact mode: $R_{sc} = (R_s + R_I) > R_s$. This latter situation is manifested in the EECs shown in Figures 4B and 5B.

S.6. Effects of Citric Acid Complexing Agent on Potentiodynamic Polarization Plots

Figure S3 compares potentiodynamic polarization plots recorded for a Mo test sample in a selected set of CMP slurries (a) without and (b) with the inclusion of CA as a complexing agent. The general slurry composition is indicated at the top of the figure, and the specific variations of this composition are noted in the different panels. The plots collected in the CMP sample's stationary hold (H) and active polish (P) arrangements are shown in the left and right columns of the figure, respectively. The plots (a) in Figure S.3 indicate the current densities (strengths) of faradaic steps which are largely dictated by those of reaction (7), and are supported in the presence of surface films with the predominant makeup of (a) $MoO_3.2H_2O/MoO_3$ [plots (a)] or $MoH_{-1}Cit(OH)_2$ [plots (b)].

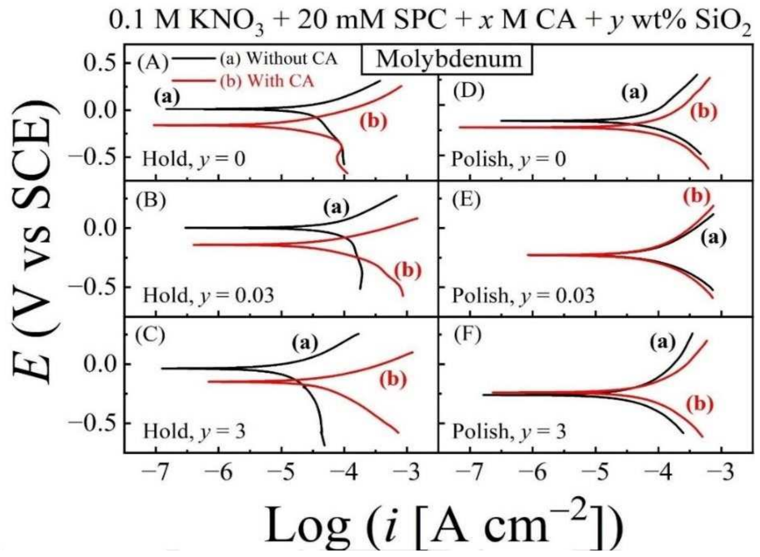

Figure S.3. Effects of CA on (iR_s corrected) polarization plots for a molybdenum sample examined under polishing in weakly alkaline CMP slurries. The concentrations of different additives in the slurries are indicated in the figure.

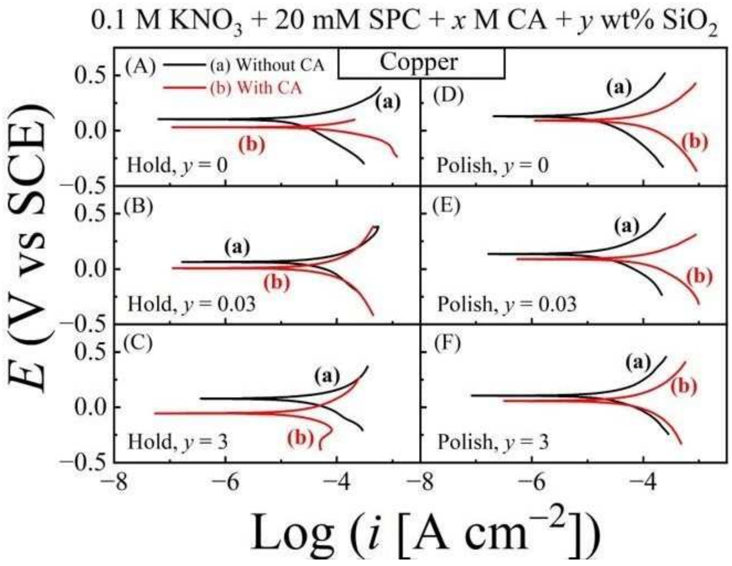

Figure S.4. Effects of CA on (iR_s corrected) polarization plots for a copper sample examined under polishing in weakly alkaline CMP slurries. The concentrations of different additives in the slurries are indicated in the figure. .

Figure S.4 shows potentiodynamic polarization data for the Cu CMP sample where the plots for the different experimental conditions are arranged and labeled using the same format as that used in Figure S3. These results for Cu and their implications are also like those observed for Mo in Figure S.3. Overall, the surface activity of Cu is augmented in the presence of CA where the $(CuCit_2H_{-2})^{4-}$ complex is formed at the Cu surface. The relatively higher cathodic currents seen for plot (b) in Figure S.3C has been explained in the context of Figure 9F.

Under mechanical abrasion, the surface films on a CMP surface generally acquire an equilibrium coverage (ideally near zero) as the rate of film formation by chemical effects is balanced with the rate of film removal by the mechanical action of the polishing pad and abrasive particles. The data shown in the right column of Figure S4 corresponds to this situation. Here once again, the surface activity of the Cu CMP sample is favored in the presence of CA.

S.7. Additional Details of CNLS-Analyzed Impedance Elements

The CNLS analyzed impedance variables obtained from the EIS data in Figures 4A and 5A are listed with their values in Tables 2 and 3, respectively. The calculated uncertainties for each of the fitted elements are presented here in Tables S1 and S2. The notations used here to list the impedance elements, as well as their functions in the present experiments have been introduced in the main article. As indicated in Tables S1 and S2, the percentage errors in all the CNLS calculated impedance elements were essentially limited under 10%.

It is also useful to note that, in the limit, $\omega \to \infty$, the total impedance of the EEC in Figure 4B becomes $Z \approx R_{sc}$, while at $\omega \to 0$, one finds $Z \approx R_{sc} + R_{p1} + R_{p2}$. At the corresponding frequency limits, the impedance of the EEC in Fig 5B take becomes R_s and R_p, respectively. According to these results, both EECs in Figure 4B and 5B meet the EIS validation criterion of finite impedance.

To fit plots (a), (b) and (c) in Figure 4A, it was necessary to set: a = 0.5 in the above formula of $Z(Q_a)$, which led to another special case of the CPE impedance, namely the semi-infinite Warburg impedance, W_a, which describes diffusion of OH^- (and Cit^{3-}, when present) from the slurry to the anion-adsorption sites. Thus, for plots (a), (b) and (c) in Figure 4A, the CPE impedance takes the form of a diffusion impedance:

$$Z(Q_a) \equiv Z\,(W_a) = \frac{1}{y_a (j\omega)^{0.5}} = \frac{(1\text{-}j)\sigma_a}{\sqrt{\omega}} \qquad \text{(S.2)}$$

which follows from the identity, $(j)^{-1/2} = (1-j)/\sqrt{2}$, and the definition, $\sigma_a = 1/(\sqrt{2}y_a)$. Here σ_a denotes the frequency independent amplitude of Warburg impedance for semi-infinite diffusion in the solution phase.

Table S.1. Fitting Errors for Impedance Parameters Obtained by CNLS Fitting EIS Data in Figure 4A for Molybdenum

CMP Parameter (Unit)	Percentile Errors in Impedance Parameter (%) CMP Systems					
	a	b	c	d	e	f
R_{sc} (Ω cm^{-2})	0.05	0.07	0.09	0.06	0.65	0.28
Y_d (μS s^a cm^{-2})	2.49	3.18	3.03	ND	ND	ND
d	0.62	0.79	0.98	ND	ND	ND
C_d (μF cm^{-2})	ND	ND	ND	6.93	0.90	3.79
R_{p1} (Ω cm^{-2})	1.03	1.14	1.66	8.84	0.32	6.73
R_{p2} (Ω cm^{-2})	ND	ND	ND	2.09	1.26	2.36
σ_a (Ω s$^{-0.5}$)	0.97	1.02	4.17	ND	ND	ND
C_a (μF cm^{-2})	ND	ND	ND	11.61	2.18	2.84

The CPE variables d and a, included in both Tables S1 and S2, can have values between the values, 0 and 1; if any of these two parameters acquires the value of 1, the corresponding CPE transforms to a simple capacitance. This latter situation applies to Q_d as well as Q_a for Nyquist plot (d), (e), and (f) in Figure 4A. In these latter cases, d =1, $Q_d \equiv C_d$ (double layer capacitance), a =1, and $Q_a \equiv C_a$ (adsorption capacitance), so that $Z(Q_d) \equiv Z(C_d) = 1/(j\omega C_d)$ and $Z(Q_a) \equiv Z(C_a) = 1/(j\omega C_a)$. The impedance elements considered in Figure 5B are comparatively more standard for a modified Randles EEC often detected for metal CMP systems.

In Figure 4A, the high-frequency (left side) Nyquist intercepts on the Z' axis represents the slurry resistances, R_{sc}, measured in sample-pad contact for the Mo sample. With increasing [SiO_2] in the slurry, the electrically insulating abrasives occupy larger volumes of the pad-sample interfacial fluid, and block electrochemically active surface sites of Mo by adsorption. As a result, the interception points of Nyquist plots on the Z' axis correspondingly shift to higher values. As the slurry conductivity increases with the inclusion of CA, plots (d), (e) and (f) are shifted toward lower values of Z' with respect to plots (a), (b) and (c). Owing to the same mechanism, the Nyquist plots for Cu in Fig. 5A also move to higher values of Z' as [SiO_2] is increased in the slurry and R_{sc} increases.

Table S.2. Fitting Errors for Impedance Parameters Obtained by CNLS Fitting EIS Data in Figure 5A for Copper

CMP Parameter (Unit)	Percentile Errors in Impedance Parameter (%) CMP Systems					
	a	b	c	d	e	f
R_{sc} (Ω cm^{-2})	0.66	0.25	0.76	0.31	0.14	0.27
Y_d (μS s^a cm^{-2})	ND	ND	ND	1.87	2.03	1.90
d	ND	ND	ND	0.69	0.45	0.74
C_d (μF cm^{-2})	8.74	2.98	13.95	ND	ND	ND
R_a (Ω cm^{-2})	2.85	3.05	2.98	7.79	4.31	4.13
Y_a (μS s^a cm^{-2})	1.00	1.83	1.02	ND	ND	ND
a	0.29	0.45	0.32	ND	ND	ND
C_d (μF cm^{-2})	ND	ND	ND	5.73	4.46	3.28
R_p (Ω cm^{-2})	0.22	0.56	0.29	0.28	0.29	0.34

References

1. Lai, J.-Y.; Saka, N.; Chun, J.-H. Evolution of copper-oxide damascene structures in chemical mechanical polishing: II. Copper dishing and oxide erosion. *Journal of The Electrochemical Society* **2001**, *149*, G41.
2. Zhang, B.; Liu, Y.; Wang, C. BTA free alkaline slurries developed for copper and barrier CMP. *ECS Journal of Solid State Science and Technology* **2015**, *4*, P5112.
3. Song, T.; Lee, K.; Sun, S.; Ko, H.; Cho, S.B.; Lee, G.; Lee, D.; Paik, U. Modulation of Molybdenum Oxidation State Via Catalytic-Oxidation. *Appl. Surf. Sci. 615*, 156330
4. Saji, V.S.; Lee, C.W. Molybdenum, molybdenum oxides, and their electrochemistry. *ChemSusChem* **2012**, *5*, 1146-1161.
5. Feng, H.; Cao, L.-A.; Feng, J.-Y.; Qu, X.-P. Effects of Colloidal Silica on the CMP of Molybdenum in the Alkaline Slurry. In Proceedings of the Proceedings of International Conference on Planarization/CMP Technology 2014, 2014; pp. 66-69.
6. Milazzo, G.; Caroli, S. *Tables of Standard Electrode Potentials*; John Wiley: New York, 1978.
7. Patri, U.B.; Aksu, S.; Babu, S.V. Role of the Functional Groups of Complexing Agents in Copper Slurries. *Journal of The Electrochemical Society* **2006**, *153*, G650-G659, doi:10.1149/1.2199307.
8. Wei, S.; Roy, D. Galvanodynamic probing of tribologically assisted material removal under chemical control: A cobalt/copper case study for application in chemical mechanical planarization. *Tribology International* **2023**, *179*, 108185.
9. Gamagedara, K.; Roy, D. Experimental Strategies for Studying Tribo-Electrochemical Aspects of Chemical–Mechanical Planarization. *Lubricants* **2024**, *12*, 63.
